# Conserved Molecular Underpinnings and Characterization of a Role for Caveolin-1 in the Tumor Microenvironment of Mature T-Cell Lymphomas

**DOI:** 10.1371/journal.pone.0142682

**Published:** 2015-11-13

**Authors:** Tyler A. Herek, Timothy D. Shew, Heather N. Spurgin, Christine E. Cutucache

**Affiliations:** Department of Biology, University of Nebraska at Omaha, Omaha, Nebraska, United States of America; University of British Columbia, CANADA

## Abstract

Neoplasms of extra-thymic T-cell origin represent a rare and difficult population characterized by poor clinical outcome, aggressive presentation, and poorly defined molecular characteristics. Much work has been done to gain greater insights into distinguishing features among malignant subtypes, but there also exists a need to identify unifying characteristics to assist in rapid diagnosis and subsequent potential treatment. Herein, we investigated gene expression data of five different mature T-cell lymphoma subtypes (n = 187) and found 21 genes to be up- and down-regulated across all malignancies in comparison to healthy CD4^+^ and CD8^+^ T-cell controls (n = 52). From these results, we sought to characterize a role for *caveolin-1* (*CAV1*), a gene with previous description in the progression of both solid and hematological tumors. *Caveolin-1* was upregulated, albeit with a heterogeneous nature, across all mature T-cell lymphoma subtypes, a finding confirmed using immunohistochemical staining on an independent sampling of mature T-cell lymphoma biopsies (n = 65 cases). Further, stratifying malignant samples in accordance with high and low *CAV1* expression revealed that higher expression of *CAV1* in mature T-cell lymphomas is analogous with an enhanced inflammatory and invasive gene expression profile. Taken together, these results demonstrate a role for *CAV1* in the tumor microenvironment of mature T-cell malignancies and point toward potential prognostic implications.

## Introduction

Mature T-cell lymphomas are a heterogeneous group of malignancies representing 10–15% of all non-Hodgkin’s lymphomas with 17,850 cases diagnosed in the United States between 2003–2012 [1, 2]. Mature T-cell lymphomas are characterized by aggressive growth, generally poor clinical outcome, and only a paucity of reported genetic abnormalities [3–6]. Currently, the World Health Organization recognizes a number of mature T-cell lymphoma subtypes, including: angioimmunoblastic T-cell lymphoma (AITL), anaplastic large cell lymphoma (ALCL), adult T-cell leukemia/lymphoma (ATLL), hepatosplenic T-cell lymphoma (HSTL), and peripheral T-cell lymphoma, not otherwise specified (PTCL-NOS) [7].

The state of research on mature T-cell lymphomas aims to enhance the recognition of molecular subtypes, thereby improving diagnostics; ultimately these produce improved prognostic models to aide in treatment [8–13]. Advances in the area of diagnostics lead to increased classification rates diverging from PTCL-NOS [8, 10, 11], which has been considered a “wastebasket” category [7]. While molecular diagnostics to improve the classification rates of T-cell lymphoma subtypes have obvious value in terms of targeted treatment, understanding *unifying* characteristics of a group of malignancies sharing an extra-thymic cell-of-origin is warranted. Therefore, an enhanced understanding of the shared molecular underpinnings of neoplasms of T-cell origin could lead toward the development of novel combinatorial therapies and information regarding the basic biology of mature T-cell lymphomas.

The goal of this study was to conduct an expansive meta-analysis (sometimes termed mega-analysis) [14] of microarray data on mature nodal and splenic T-cell lymphomas to construct a gene signature shared across all subtypes. To this end, we mined the NCBI GEO DataSets (Table 1) for chip-matched, mature T-cell lymphoma samples (n = 187) and healthy CD4^+^ and CD8^+^ T-cell controls (n = 52) with focus on genes annotated to function in T-cell receptor signaling, T-cell co-stimulation, T-cell homeostasis, and T-cell differentiation in the gene ontology (GO) directory to mitigate background from the stromal compartment. The abovementioned genetic findings were then corroborated at the protein level using human biopsies of mature T-cell lymphoma cases (n = 130 core biopsies from n = 65 unique cases).

**Table 1:** Publically available, chip-matched GEO DataSets of mature T-cell lymphomas and healthy CD4^+^ and CD8^+^ T cells utilized for gene expression profiling.

GEO Accession	Samples Utilized (n-value)	PMID
GSE43017	Healthy Samples: CD4^+^ T cell (5)	N/A
GSE6338	Malignant Samples: PTCL-NOS (28), AITL (6), ALCL (6) Healthy Samples: CD4^+^ T cell (5), CD8^+^ T cell (5),	17304354
GSE61399	Healthy Samples: CD4^+^ T cell (3)	N/A
GSE14879	Malignant Samples: ALCL (9)	19657361, 24376854
GSE19067	Malignant Samples: HSTL (4)	21052088
GSE19069	Malignant Samples: AITL (37), ALCL (30), ATLL (13), PTCL-NOS (50) Healthy Samples: CD4^+^ T cells (1)	19965671
GSE25087	Healthy Samples: CD4^+^ T cell (3)	21164017
GSE31773	Healthy Samples: CD4^+^ T cell (8), CD8^+^ T cell (8)	21917308
GSE36769	Healthy Samples: CD4^+^ T cell (4)	23778140
GSE49954	Healthy Samples CD4^+^ T cell (5), CD8^+^ T cell (5)	24130824
GSE57944	Malignant Samples: HSTL (4)	25057852

AITL, angioimmunoblastic T-cell lymphoma; ALCL, anaplastic large cell lymphoma; ATLL, adult T-cell leukemia/lymphoma; HSTL, hepatosplenic T-cell lymphoma; PTCL-NOS, peripheral T-cell lymphoma, not otherwise specified; PMID, PubMed identifier

Herein, presented are the findings of the genetic analysis with an increased focus on *caveolin-1* (*CAV1*), a gene seen heterogeneously upregulated across all T-cell lymphoma subtypes, mimicking data observed in chronic lymphocytic leukemia, a B-cell neoplasm [15, 16, 17]. These data support a correlation between *CAV1* and the promotion of an inflammatory and invasive phenotype in mature T-cell lymphomas. Future research will be required to determine whether CAV1 is directly involved in the process.”

## Results

### Construction of a shared T-cell compartment gene signature across mature T-cell lymphomas

**Fig 1:**
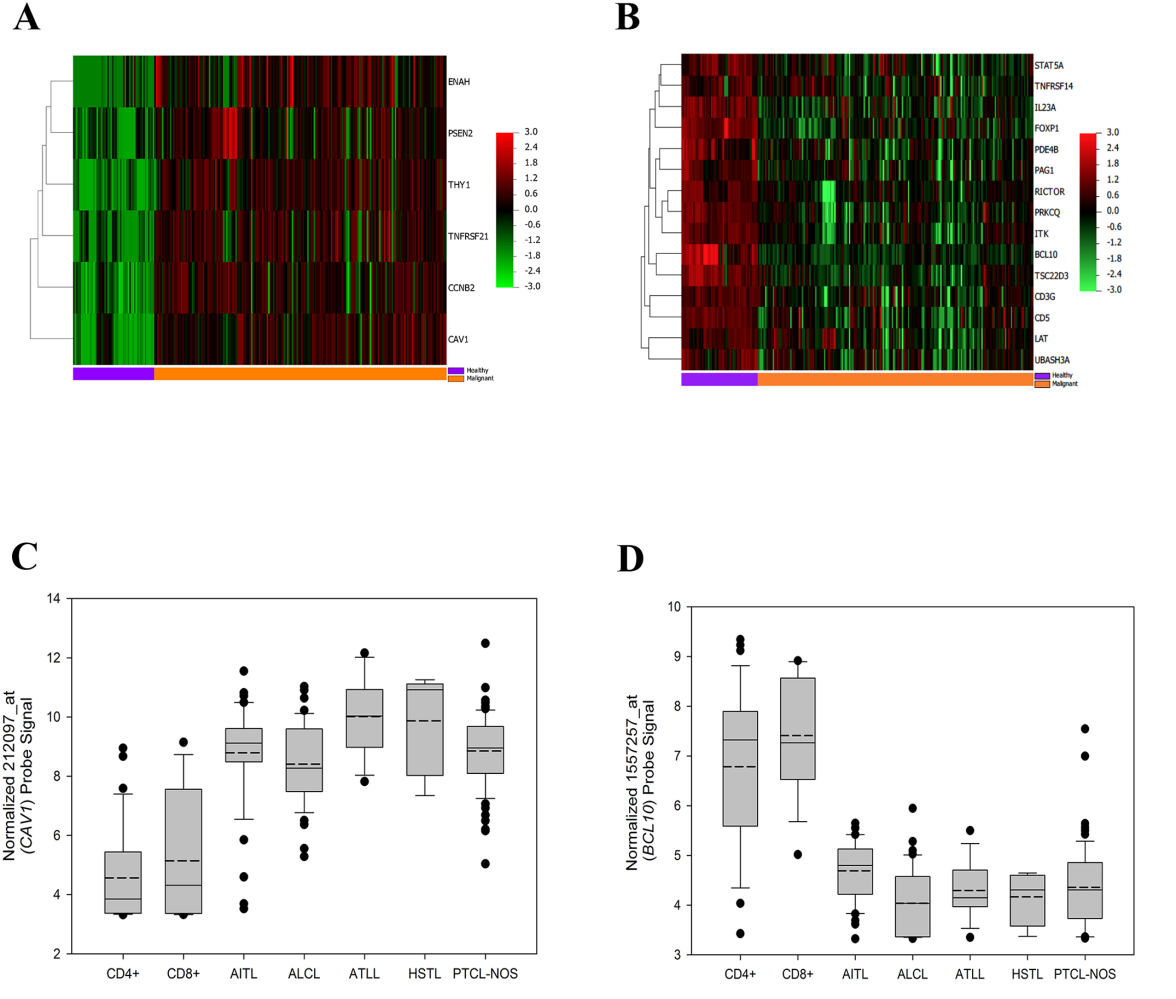
Construction of a shared T-cell compartment gene signature across mature T-cell lymphomas. (A) Heatmap of genes (n = 6) significantly (p < 0.001, FDR < 0.001) upregulated across all T-cell lymphoma subtypes as compared with healthy controls. Genes are mean-centered, standard-deviation scaled, and clustered according to average linkage. Samples are aligned according to healthy (purple bar) or malignant (orange bar) designations. (B) Heatmap of genes (n = 15) significantly (p < 0.001, FDR < 0.001) downregulated across all T-cell lymphoma subtypes as compared to healthy controls. Genes are mean-centered, standard-deviation scaled, and clustered according to average linkage. Samples are aligned according to healthy (purple bar) or malignant (orange bar) designations. (C) Boxplot of normalized 212097_at (*CAV1*) probe signal across sample types. Continuous line represents median value of sample, discontinuous line represents mean value. (D) Boxplot of normalized 1557257_at (*BCL10*) probe signal for representation. Continuous line represents median value of sample, discontinuous line represents mean value.

In order to delineate a shared T-cell compartment signature among a diverse grouping of mature T-cell lymphomas, we conducted differential expression analyses of the five different mature T-cell lymphoma subtypes collected, focusing on GO annotations specific to T-cell biology. We analyzed each T-cell lymphoma subtype separately, with a final manual compilation of genes found to be differentially expressed across all subtypes. This analysis revealed an up-regulation of 6 genes (namely *CAV1*, *CCNB2*, *ENAH*, *PSEN2*, *THY1*, *TNFRSF21*) (Fig 1A) and a down-regulation of 15 genes (namely *BCL10*, *CD3G*, *CD5*, *FOXP1*, *IL23A*, *ITK*, *LAT*, *PAG1*, *PDE4B*, *PRKCQ*, *RICTOR*, *STAT5A*, *TNFRSF14*, *TSC22D3*, *UBASH3A*) (Fig 1B) that were observed to be differentially expressed (p < 0.001, FDR < 0.001) in all T-cell lymphoma subtypes (Table 2). Further, 52 genes were found to either be up- or down-regulated in at least 3 out of 5 of the analyzed subtypes (S1 Table).

**Table 2:** Genes significantly up- or down-regulated across all mature T-cell lymphoma subtypes as compared with healthy CD4^+^ and CD8^+^ T-cell controls.

Gene Name	Symbol	Chromosomal Location	AITL Fold-Change	ALCL Fold-Change	ATLL Fold-Change	HSTL Fold-Change	PTCL-NOS Fold-Change
Caveolin-1	CAV1	7q31.1	16.33	12.52	38.17	34.41	16.33
Cyclin B2	CCNB2	15q22.2	7.55	8.93	13.99	9.65	7.55
Thy-1 cell surface antigen	THY1	11q23.3	13.65	12.98	9.46	2.46	13.65
Tumor necrosis factor receptor superfamily member 21	TNFRSF21	6p21.1	6.3.8	6.27	5.34	9.30	6.38
Enabled homolog	ENAH	1q42.12	5.62	5.36	2.52	22.63	5.62
Presenilin 2	PSEN2	1q42.13	2.94	4.77	2.26	3.39	2.94
3g molecule, gamma (CD3-TCR complex)	CD3G	11q23	-3.01	-6.81	-1.65	-2.02	-3.00
Tumor necrosis factor receptor superfamily member 14	TNFRSF14	1p36.32	-1.49	-1.58	-1.69	-1.99	-1.49
RPTOR independent companion of MTOR, complex 2	RICTOR	5p13.1	-2.42	-4.36	-1.75	-2.25	-2.42
Linker for activation of T-cells	LAT	16p11.2	-2.91	-3.61	-1.92	-3.76	-2.91
CD5 molecule	CD5	11q13	-4.12	-6.55	-2.10	-17.27	-4.11
Interleukin 23, alpha subunit p19	IL23A	12q13.3	-3.26	-3.50	-2.23	-4.51	-3.26
IL2-inducible T-cell kinase	ITK	5q31-q32	-3.67	-10.78	-2.36	-2.70	-3.67
Signal transducer and activator of transcription 5A	STAT5A	17q11.2	-2.14	-2.11	-2.50	-2.63	-2.14
TSC22 domain family, member 3	TSC22D3	Xq22.3	-5.66	-6.63	-2.56	-4.07	-5.66
Ubiquitin-associated and SH3 domain-containing protein A	UBASH3A	21q22.3	-2.43	-4.27	-2.86	-6.97	-2.43
Protein kinase C theta	PRKCQ	10p15	-2.98	-8.26	-3.58	-2.92	-2.98
Forkhead box P1	FOXP1	3p14.1	-4.90	-3.96	-3.69	-3.40	-4.90
Phosphoprotein membrane anchor with glycosphingolipid microdomains 1	PAG1	8q21.13	-3.81	-2.32	-4.30	-3.69	-3.81
B-cell CLL/lymphoma 10	BCL10	1p22	-4.96	-7.82	-6.54	-7.15	-4.96
Phosphodiesterase 4B, cAMP-specific	PDE4B	1p31	-4.21	-3.55	-7.44	-3.42	-4.21

AITL, angioimmunoblastic T-cell lymphoma; ALCL, anaplastic large cell lymphoma; ATLL, adult T-cell leukemia/lymphoma; HSTL, hepatosplenic T-cell lymphoma; PTCL-NOS, peripheral T-cell lymphoma, not otherwise specified

To show the robust nature of these findings, the 21 pan-regulated genes were used for class prediction using the top-scoring pairs (TSP) method [18]. This pairwise comparison method selects a family of *gene pairs* and classifies samples based on a decision involving the comparison of the ratio of mRNA abundance for selected gene pairs [18]. Of the 21 candidate genes, TSP scored the expression ratio of *CAV1* (Fig 1C) and *BCL10* (Fig 1D) to hold the greatest magnitude of change. Using the *CAV1-BCL10* classifier, T-cell lymphoma samples were classified with 98.4% sensitivity and 88.5% specificity (S2 Table). These results suggest that the inverse expression of *CAV1* and *BCL10* may assist in the diagnosis of mature T-cell lymphomas, and can be attributable to all analyzed subtypes.

### Stratification of mature T-cell lymphomas by expression of *Caveolin-1*

**Fig 2:**
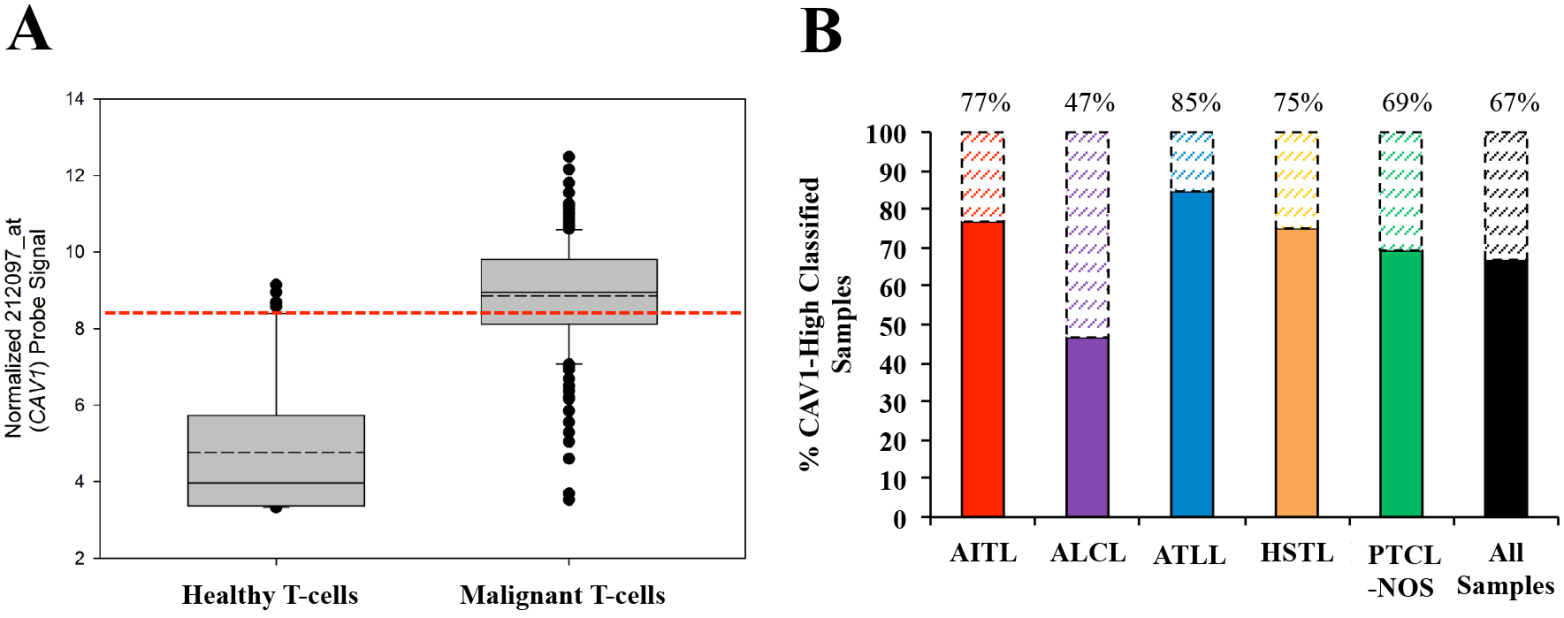
T-cell lymphomas can be divided into *CAV1*-High and *CAV*-1 Low subgroups. (A) Boxplot of normalized 212097_at (*CAV1*) probe signal between pooled healthy controls and malignant samples. Continuous line represents median value, discontinuous line represents mean. Red line represents threshold for *CAV1*-High or *CAV1*-Low expression determination and is set 2 standard deviations above the healthy T-cell mean value. (B) Classification of *CAV1*-High and *CAV1*-Low samples according to expression threshold. Solid bars and above-bar values represent percentage of *CAV1*-High samples per subtype. 67% (125/187) of all malignant samples were classified as *CAV1*-High. 77% (33/43) of AITL samples, 47% (21/45) of ALCL samples, 85% (11/13) of ATLL samples, 75% (6/8) of HSTL samples, 69% (54/78) of PTCL-NOS samples.

As previously reported, higher *CAV1* expression in B-cell lymphomas leads to a more aggressive disease [15, 16] with an inferior overall survival among patients with higher *CAV1* expression [15]. Therefore, we decided to further investigate the differences between *CAV1*-High and *CAV1*-Low expressing mature T-cell lymphoma samples in an effort to characterize the impact of CAV1 on the tumor microenvironment (TME). Analysis of *CAV1* expression using 212097_at probe signal intensity revealed that all mature T-cell lymphoma subtypes analyzed have a higher mean expression of *CAV1* (p < 0.001, FDR < 0.001) as compared to healthy CD4^+^ and CD8^+^ T-cell controls (Fig 1C). However, as expected, there exists a heterogeneous expression of *CAV1* amongst the malignant samples. To stratify samples into the abovementioned groups, healthy controls and malignant samples were pooled, respectively. Moreover, a *CAV1*-Low expression threshold was calculated based on the distribution of *CAV1* expression in healthy samples (Fig 2A). Using this threshold, 67% (125/187) of mature T-cell lymphoma samples were classified as *CAV1*-High (Fig 2B). For each T-cell lymphoma subtype: 77% (33/43) of AITL samples, 47% (21/45) of ALCL samples, 85% (11/13) of ATLL samples, 75% (6/8) of HSTL samples, and 69% (54/78) of PTCL-NOS samples were classified as *CAV1*-High.

### The *CAV1*-High tumor microenvironment is enriched in genes involved in the immune response, chemokine/cytokine activity, and invasive genes

As shown above in Fig 2, mature T-cell lymphoma samples highly express *CAV1*, with 67% of samples harboring expression that is 2 standard deviations above mean *CAV1* expression in CD4^+^ and CD8^+^ T cells. However, 33% of samples express *CAV1* within a healthy range, with no one-subtype exclusively harboring high *CAV1* expression. To investigate the impact of higher *CAV1* expression on the TME (i.e. the whole tissue sample), we used the class comparison among groups of arrays method. To expand our analysis to the TME, yet reduce inter-malignancy variation, only genes significantly correlated to *CAV1* expression were considered (see Materials and Methods). Of the 7,170 genes analyzed, we report that 2,624 genes were differentially expressed (p < 0.001, FDR < 1% at 99% confidence) between the two classes, 1,758 (67%) of which were overexpressed in the *CAV1*-High group. For analysis, we input the fold-changes values from the genes found differentially expressed into the PANTHER gene analysis tool [19, 20]. PANTHER enrichment analyses reveal the *CAV1*-High TME to be enriched in genes annotated to be involved in chemokine/cytokine activity, invasion (migration/motility/movement), and immune processes (Table 3). Chemokine/cytokine activity featured enrichments in biological processes, molecular functions, and the chemokine protein family. Invasive activity was found to harbor enrichments in biological processes and enrichment of the integrin signaling pathway (S1 Fig). Immune processes featured enrichments in biological processes. Furthermore, overrepresentation analysis revealed significant (p < 0.05) overrepresentation of genes upregulated in *CAV1*-High conditions to be involved in the PANTHER pathways for inflammation mediated by chemokine and cytokine signaling (S2 Fig) and angiogenesis (S3 Fig). As *CAV1* is overexpressed in mature T-cell lymphomas, the tumor microenvironment adopts a gene expression profile indicative of increased inflammation and metastatic potential, as demonstrated above. This phenotype suggests a correlation between *CAV1*expression and increased inflammation in the tumor microenvironment of T-cell lymphomas. Increased inflammation could lead to increased tumor burden and recruitment of non-neoplastic cells [21, 22], potentially leading to a more aggressive disease.

**Table 3:** PANTHER enrichment analysis of genes upregulated in the *CAV1*-High tumor microenvironment.

**Immune Enrichment**	**GO Annotation**	**Number of Genes**	**OverUnder**	**p-value** ^1^
*Biological Process*:	Regulation Of Leukocyte Activation	59	+	2.10E-02
	Activation Of Immune Response	65	+	3.32E-02
	Immune Effector Process	76	+	1.03E-04
	Positive Regulation Of Immune Response	81	+	2.61E-02
	Regulation Of Immune Response	125	+	4.92E-02
	Regulation Of Immune System Process	189	+	2.85E-04
	Immune Response	194	+	1.00E-04
**Chemokine/Cytokine Enrichment**	**GO Annotation**	**Number of Genes**	**OverUnder**	**p-value**
*Biological Process*:	Cell Chemotaxis	24	+	9.08E-05
	Regulation Of Chemotaxis	30	+	2.81E-02
	Cytokine-Mediated Signaling Pathway	65	+	1.33E-03
	Inflammatory Response	68	+	1.21E-02
	Cellular Response To Cytokine Stimulus	86	+	7.25E-05
	Response To Cytokine	106	+	3.97E-06
*Molecular Function*:	Chemokine Activity	11	+	2.79E-03
	Chemokine Receptor Binding	12	+	1.11E-03
*Protein Family*:	Chemokine	9	+	3.03E-03
**Invasion Enrichment**	**GO Annotation**	**Number of Genes**	**OverUnder**	**p-value** ^1^
*Biological Process*:	Regulation Of Leukocyte Migration	23	+	4.32E-02
	Extracellular Matrix Organization	72	+	2.88E-03
	Regulation Of Cell Migration	98	+	8.69E-04
	Regulation Of Cell Motility	104	+	7.91E-04
	Regulation Of Cellular Component Movement	112	+	5.30E-04
*Pathway*:	Integrin Signaling Pathway	36	+	4.44E-02

^1^ p-values calculated using the Bonferroni correction

### Immunohistochemical analysis on an independent sampling of mature T-cell lymphomas

**Fig 3:**
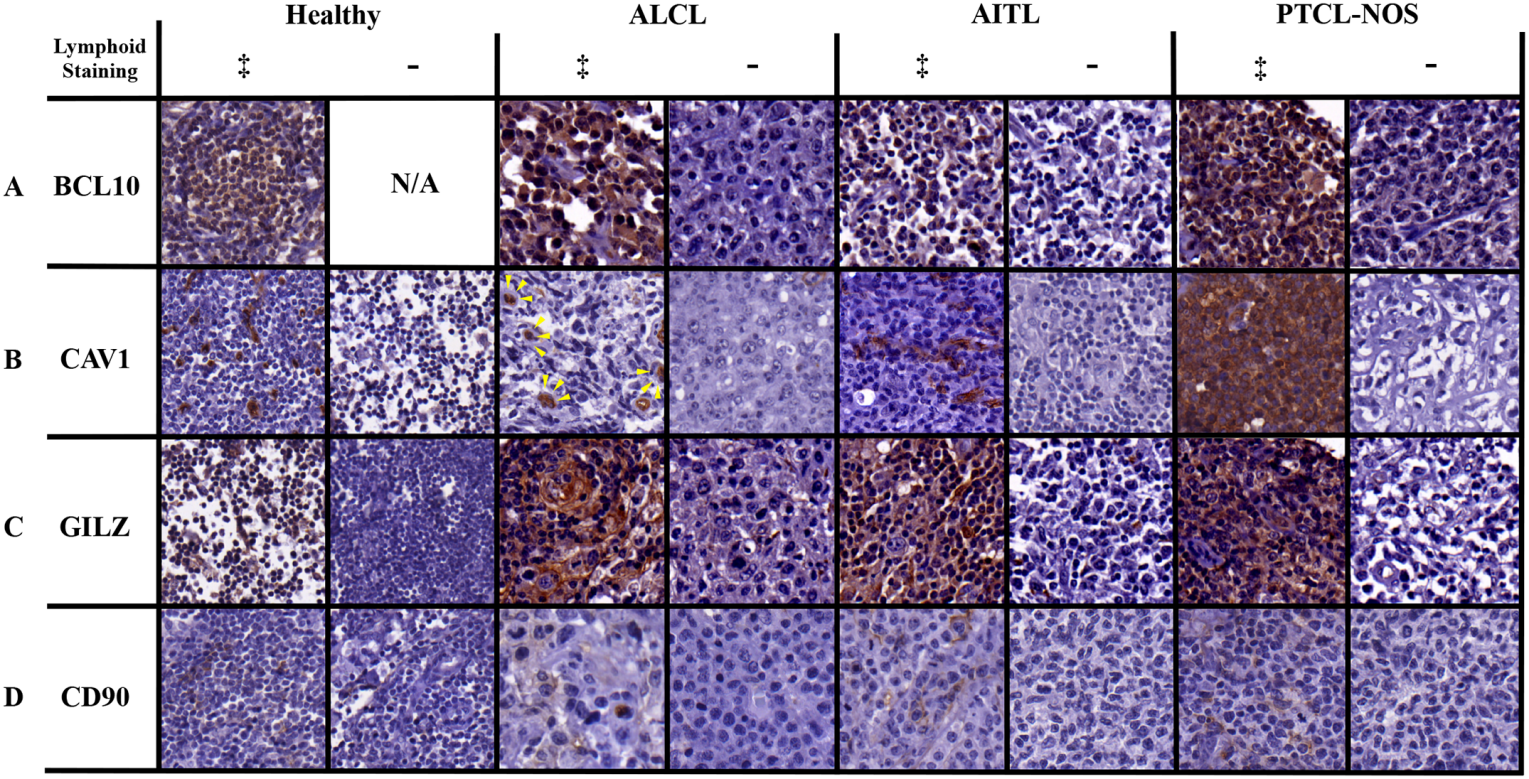
Immunohistochemical staining of mature T-cell lymphoma biopsies. Representative positive and negative lymphoid-staining samples for BCL10, CAV1, GILZ, and CD90 are presented. All images were taken at 60x magnification. No healthy lymph nodes were negative for BCL10 lymphoid staining. Yellow arrowheads in ALCL positive sample point toward punctate CAV1 staining in hallmark horse-shoe nuclei tumor cells.

To investigate our genetic findings at the protein level, we purchased LY6161 high-density lymphoma and normal lymph node tissue arrays from U.S. Biomax, Inc. These tissue arrays contained n = 25 normal lymph nodes and n = 130 core biopsies from n = 65 unique, mature T-cell lymphoma patients (8 AITL, 14 ALCL, and 43 PTCL-NOS patients, respectively). Separate tissue array slides were stained with antibodies directed towards: BCL10, CAV1, CD90 (Thy-1), GILZ (TSC22D3), in addition to standard hematoxylin and eosin staining. Summarized analysis of immunohistochemical staining can be found in Table 4, detailed analysis of individual samples can be found in S3 Table, with representative imaging found in Fig 3.

**Table 4:** Summary of diagnoses, patient characteristics, and staining properties of T-cell lymphoma histological samples from the Ly6161 tissue microarray from U.S. Biomax, Inc.

Diagnosis	Sample #	Median Age^1^	% Male	BCL10^2^	CAV1^2^	GILZ^2^	CD90^2^
Anaplastic large cell lymphoma	14	45	50% (7/14)	93% (13/14)	43% (6/14)	93% (13/14)	36% (5/14)
Angioimmunoblastic T-cell lymphoma	8	50	63% (5/8)	88% (7/8)	63% (5/8)	100% (8/8)	75% (6/8)
Peripheral T-cell lymphoma-NOS	43	41	53% (23/43)	91% (39/43)	58% (25/43)	93% (40/43)	42% (18/43)

^1^ Age reported in years

^2^ Percentages reflect staining observed in lymphoid cells

For BCL10 staining, 88% (7/8) of AITL samples, 93% (13/14) of ALCL samples, and 91% (39/43) PTCL-NOS samples were scored positive for lymphoid staining. 2 ALCL samples and 1 PTCL-NOS sample were scored light in comparison to other positive samples in terms of lymphoid staining. 1 PTCL-NOS sample was scored with light stromal/non-lymphoid staining, and 1 AITL sample, 1 ALCL sample, and 3 PTCL-NOS samples were scored negative for lymphoid staining. For CAV1 staining, 63% (5/8) of AITL samples, 43% (6/14) of ALCL samples, and 58% (25/43) of PTCL-NOS samples were scored positive for lymphoid staining. 1 AITL sample was negative for CAV1 staining. 2 AITL samples, 8 ALCL samples, and 18 PTCL-NOS samples were positive for stromal/non-lymphoid staining. For GILZ staining, 100% (8/8) of AITL samples, 93% (13/14) of ALCL samples, and 93% (40/43) PTCL-NOS samples were scored positive for lymphoid staining. 2 AITL samples, 1 ALCL sample, and 7 PTCL-NOS samples were scored light in comparison to other positive samples in terms of lymphoid staining. 1 ALCL sample was scored with stromal/non-lymphoid staining, and 3 PTCL-NOS samples were scored negative for presence of stain. For CD90 staining, 75% (6/8) of AITL samples, 36% (5/14) of ALCL samples, and 42% (18/43) of PTCL-NOS samples were scored positive for lymphoid staining. 6 ALCL samples and 13 PTCL-NOS samples were scored positive for stromal/non-lymphoid staining, and 2 AITL samples, 3 ALCL samples, and 12 PTCL-NOS samples were scored negative for presence of stain.

The results from protein staining of the TMAs support the genetic findings two-fold. First, as the GEP data are publically derived, routine qPCR validations are unavailable and a secondary confirmation of protein analysis is needed. The protein findings support the GEP results through reaffirming that the GEP data are originating from tumor lymphocytes within the samples and are not strictly artifacts of stromal components, as the biopsy samples used for the GEP analyses were neither flow sorted nor laser micro-dissected and we can determine the localization of staining based on histology of the TMA. These findings are supported by the staining patterns displayed in Fig 3 showing positive staining in tumor lymphocytes as opposed to solely stromal and/or non-lymphocyte staining. Secondly, as the TMA samples are completely distinct in patient origin to those used in the GEP we can see that the GEP results, this shows the strength of these findings in that they are consistent between genomic and protein expression across a large sample size of cases of lymphoma.

### Long non-coding RNA 273 is computationally predicted to interact with members of a CAV1-interaction network

**Fig 4:**
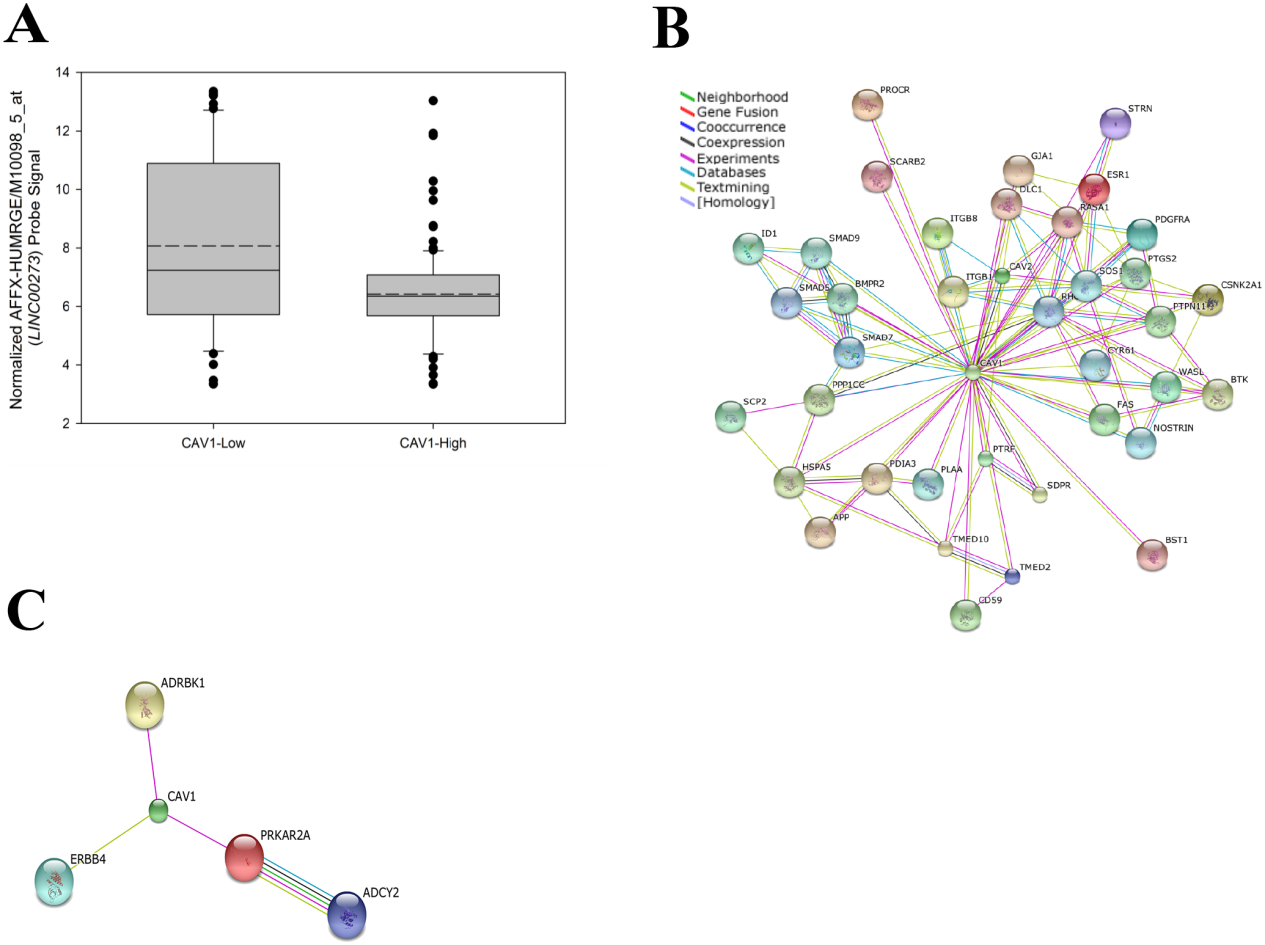
Long non-Coding RNA 273 is computationally predicted to interact with members of a CAV1-interaction network. (A) Boxplot of normalized AFFX-HUMRGE/M10098_5_at (*LINC00273*) probe signal for *CAV1*-Low and *CAV1*-High expression groups. Continuous line represents median value, discontinuous line represents mean value. (B) CAV1-interacting proteins upregulated in the *CAV1*-High expression group. Interaction network viewed in String evidence view, connection lines are defined by in-Fig box. (C) CAV1-interacting proteins upregulated in the *CAV1*-Low expression group. Interaction network viewed in String evidence view, connection lines are defined by in-Fig box.

Strikingly, an uncharacterized long, intergenic non-coding RNA (*LINC00273*) was found to be expressed 3-fold higher in *CAV1*-Low T-cell lymphoma samples compared to *CAV1*-High (Fig 4A). As long non-coding RNAs have been implicated in RNA-protein binding complexes, we sought to investigate if *LINC00273* represented a potential RNA-protein interaction (RPI) partner with CAV1 and CAV1-interacting proteins, as *CAV1* expression was the basis upon which the abovementioned groups were stratified. Using STRING v10, we determined the predicted CAV1-interactions networks for genes upregulated in both *CAV1*-High (Fig 4B) and *CAV1*-Low groups (Fig 4C). Of the 1,758 genes upregulated in *CAV1*-High samples, 38 proteins were modeled to interact (either direct, or indirect) with CAV1 (Fig 4B). The *CAV1*-High interaction network featured 100 protein-protein interactions, of which the protein constituents were found to be enriched in GO biological processes for regulation of cell differentiation (p = 2.7x10^−4^), cell migration (p = 4.2x10^−4^), cell proliferation (p = 7.2x10^−4^), apoptotic processes (p = 2.87x10^−4^), and the KEGG TGF-beta signaling pathway (p = 1.9x10^−6^). Conversely, only 4 proteins of the 866 genes upregulated in *CAV1*-Low samples were modeled to interact with CAV1 (Fig 4C). This protein network featured 4 protein-protein interactions and was found to be enriched for the GO biological processes of fibroblast growth factor receptor signaling (p = 1.91x10^−4^) and ERBB signaling (p = 5.46x10^−4^).

Using available prediction methods for RPI, we sought to achieve a consensus computational prediction concerning the interaction potential of *LINC00273* in CAV1-interaction networks. The lncPro [23], RPISeq (both Random Forrest [RPISeq-RF] and Support Vector Machine [RPISeq-SVM] output) [24], and RPI-Pred [25] programs were used in the abovementioned RPI-prediction analysis. Of the 43 proteins evaluated for RPI, lncPro scored 100% of potential proteins as interacting, RPISeq-RF scored 100% as interacting, RPISeq-SMV scored 7% (3/43) as interacting, and RPI-Pred scored 100% as interacting. Detailed results can be found in Table 5. These computational data support a potential interaction between *LINC00273* and members of a CAV1-interaction network. However, the exact functional consequences of these interactions are not fully understood. Potentially, *LINC00273* may serve as a scaffold, increasing the stability or proximity of the CAV1-interacting proteins, or may hold regulatory properties over its interacting proteins.

**Table 5:** Computational predictions for RNA-protein interactions between *LINC00273* and CAV1-interacting proteins.

Protein	Symbol	UniProt Sequence ID	lncPro^1^	RPISeq-RF^2^	RPISeq-SVM^3^	RPI-Pred
Adenylate cyclase 2*	ADCY2	Q08462-1	76.6134	0.8	0.346	Interaction Possible
Adrenergic, beta, receptor kinase 1*	ADRBK1	P25098-1	91.8466	0.75	0.343	Interaction Possible
Amyloid beta (A4) precursor protein	APP	P05067-1	94.385	0.75	0.067	Interaction Possible
Bone marrow stromal cell antigen 1	BST1	Q10588-1	88.2213	0.65	0.083	Interaction Possible
Bone morphogenetic protein receptor, type II	BMPR2	Q13873-1	76.283	0.8	0.146	Interaction Possible
Bruton agammaglobulinemia tyrosine kinase	BTK	Q06187-1	84.8525	0.75	0.377	Interaction Possible
Casein kinase 2, alpha 1 polypeptide	CSNK2A1	P68400-1	74.3077	0.65	0.653	Interaction Possible
Caveolin-1	CAV1	Q03135-1	77.9051	0.6	0.138	Interaction Possible
Caveolin-2	CAV2	P51636-1	83.1315	0.6	0.219	Interaction Possible
CD59 molecule	CD59	P13987-1	82.6124	0.7	0.087	Interaction Possible
Cysteine-rich, angiogenic inducer, 61	CYR61	O00622-1	89.9187	0.7	0.424	Interaction Possible
DLC1 Rho gtpase activating protein	DLC1	Q96QB1-2	78.2561	0.85	0.205	Interaction Possible
Erb-b2 receptor tyrosine kinase 4*	ERBB4	Q15303-1	88.6078	0.8	0.22	Interaction Possible
Estrogen receptor 1	ESR1	P03372-1	82.6917	0.65	0.164	Interaction Possible
Gap junction alpha-1 protein	GJA1	P17302-1	71.9378	0.7	0.378	Interaction Possible
Heat shock 70kda protein 5	HSPA5	P11021-1	87.4218	0.65	0.163	Interaction Possible
Inhibitor of DNA binding 1, dominant negative helix-loop-helix protein	ID1	P41134-1	63.6619	0.5	0.133	Interaction Possible
Integrin, beta 1	ITGB1	P05556-1	91.4368	0.8	0.086	Interaction Possible
Integrin, beta 8	ITGB8	P26012-1	91.3547	0.8	0.155	Interaction Possible
Nitric oxide synthase trafficking	NOSTRIN	Q8IVI9-1	89.4255	0.65	0.063	Interaction Possible
Phospholipase A2-activating protein	PLAA	Q9Y263-1	74.5552	0.7	0.199	Interaction Possible
Platelet-derived growth factor receptor, alpha polypeptide	PDGFRA	P16234-1	77.8343	0.8	0.176	Interaction Possible
Polymerase I and transcript release factor	PTRF	Q6NZI2-1	78.2171	0.7	0.201	Interaction Possible
Prostaglandin-endoperoxide synthase 2	PTGS2	P35354-1	86.235	0.95	0.232	Interaction Possible
Protein C receptor, endothelial	PROCR	Q9UNN8-1	88.6295	0.75	0.1	Interaction Possible
Protein disulfide-isomerase A3	PDIA3	P30101-1	90.1417	0.85	0.583	Interaction Possible
Protein kinase, camp-dependent, regulatory, type II, alpha*	PRKAR2A	P13861-1	93.0229	0.8	0.318	Interaction Possible
Protein phosphatase 1, catalytic subunit, gamma isozyme	PPP1CC	P36873-1	63.2618	0.6	0.387	Interaction Possible
Protein tyrosine phosphatase, non-receptor type 11	PTPN11	Q06124-1	78.559	0.6	0.022	Interaction Possible
Ras homolog family member A	RHOA	P61586-1	66.3465	0.7	0.179	Interaction Possible
RAS p21 protein activator	RASA1	P20936-1	73.3972	0.65	0.263	Interaction Possible
Scavenger receptor class B, member 2	SCARB2	Q14108-1	77.3416	0.6	0.086	Interaction Possible
Serum deprivation response	SDPR	O95810-1	73.1867	0.5	0.104	Interaction Possible
SMAD family member 5	SMAD5	Q99717-1	77.3416	0.75	0.096	Interaction Possible
SMAD family member 7	SMAD7	O15105-1	75.7177	0.65	0.239	Interaction Possible
SMAD family member 9	SMAD9	O15198-1	75.7465	0.6	0.147	Interaction Possible
Son of sevenless homolog 1	SOS1	Q07889-1	91.8128	0.7	0.255	Interaction Possible
Sterol carrier protein 2	SCP2	P22307-1	72.2967	0.75	0.071	Interaction Possible
Striatin, calmodulin binding protein	STRN	O43815-1	75.5192	0.65	0.065	Interaction Possible
Transmembrane emp24 domain trafficking protein 2	TMED2	Q15363-1	78.6391	0.8	0.327	Interaction Possible
Transmembrane emp24-like trafficking protein 10	TMED10	P49755-1	89.2447	0.65	0.522	Interaction Possible
Tumor necrosis factor receptor superfamily member 6	FAS	P25445-1	92.6536	0.6	0.091	Interaction Possible
Wiskott-Aldrich syndrome-like	WASL	O00401-1	83.887	0.55	0.228	Interaction Possible

* Proteins in the *CAV1*-Low interaction network

^1^ Interactions scored positive at values ≥50

^2^ Interactions scored positive at values ≥0.5

^3^ Interactions scored positive at values ≥0.5

## Discussion

Numerous studies have identified biomarkers and molecular signatures aimed at improving the diagnosis of mature T-cell lymphomas, herein we aimed to investigate shared molecular underpinnings that can be attributed to all subtypes. Specifically, our study brings novel insights into shared gene expression patterns (and confirmed protein expression of biopsy samples) amongst a diverse group of mature T-cell lymphomas. Our analysis uncovered 21 genes that shared differential expression in comparison to healthy CD4^+^ and CD8^+^ T cells. Together, these genes can be described as contributing to an activated phenotype, while suppressing differentiation into mature, effector T cells. Additionally, shared methods of apoptotic avoidance and ablations in TCR-related expression were observed.

Regarding a shared activated phenotype for T-cell lymphomas, of the observed upregulated genes, two (*CAV1* and *ENAH*) have been implicated in cytoskeletal remodeling (and therefore, cellular migration and immune synapse formation) following TCR-ligation [15, 26]. Knockout studies of *CAV1* that investigated immune synapse formation showed disruption of the actin cytoskeleton [15] and deregulation of cell membrane polarity [27]. These studies indicated an impairment of APC:T-cell engagement, and demonstrated a fundamental role for CAV1 as a co-stimulatory molecule in immune synapse formation, though the precise mechanism remains elusive. However, the upregulation of both *CAV1* and *ENAH* could indicate an enhancement of T-cell stimulation and activation. This observation is strengthened by a pan-upregulation of *TNFRSF21* (also known as *DR6*) and *CCNB2*. TNFRSF21 is a death domain-containing receptor of the tumor necrosis factor-receptor family [28] shown to be upregulated in peripheral CD4^+^ and CD8^+^ T cells following TCR-mediated T-cell activation in a time and NF-kB/NFAT dependent manner [29]. *CCNB2* is a G2/M specific cyclin kinetically shown to be constantly upregulated throughout T cell stimulation [30].

Furthermore, the down regulation of *TSC22D3* (also known as *GILZ*), *TNFRSF14* (also known as *HVEM)*, *CD5*, and *UBASH3A* (also known as *STS-2*) are in accordance with an activated phenotype. *GILZ* (glucocorticoid-induced leucine zipper) is an NF-κB inhibitor that prevents NF-κB binding and is independent from Rel- or I-κB-related proteins [31]. Additionally, *GILZ* expression has been shown to be inversely correlated with T-cell activation [31, 32]. *HVEM* is down-regulated following T-cell activation, in concurrence with its description as a negative regulator of T-cell activation. *HVEM* ^-/-^ mice displayed a hyper responsive phenotype of enhanced proliferation and cytokine production (IFN-γ/IL-2) [33–35], a phenotype mirrored in *STS-2* ^*-/-*^ T cells [36]. *CD5* has a demonstrated role in TCR-signaling wherein it acts as a negative regulator through an immunoreceptor tyrosine-based inhibitory motif (ITIM)-like motif [37]. The absence of *CD5* in thymocytes induces a hyper-responsive state following TCR-stimulation [38]. *CD5* expression is routinely examined during histological staining of T-cell lymphomas, and frequently harbors aberrant expression [13, 39, 40].

While displaying gene expression in accordance with an activated phenotype, all mature T-cell lymphoma samples additionally differentially regulated genes involved in effector T-cell differentiation (*GILZ*, *TNFRSF21*, *STAT5A*, *RICTOR*), T-cell homeostasis (*FOXP1* and *HVEM*), and upregulating an immature thymocyte marker (*Thy-1*/*CD90*) [41]. The upregulation of *TNFRSF21* and downregulation of *GILZ*, *STAT5A*, and *RICTOR* suggests a subversion of Th2-effector differentiation as T cells displaying similar phenotypes harbored impairment of Th2-cyotkine production or population homeostasis [42–45]. This observation suggests a potential skewing of the neoplastic T cells towards other T-cell subsets or keeping them in an undifferentiated state. For example, the cellular origin of AITL has been well studied and established as follicular helper T cells [46–48]. Despite the established plasticity among helper T-cell subsets, inter-subset regulation (i.e. maturation into one effector subset blocking the machinery required for differentiation into other subsets) occurs regularly, and has been well described [49]. Taken together, these data suggests that mature T-cell lymphomas display gene expression characteristics that subvert certain aspects of the Th2 effector phenotype. This indicates that Th2 effector T cells may not represent a widespread cell-of-origin attributable to the mature T-cell lymphoma subtypes analyzed. However, recent studies have shown that upregulation of *GATA3*, the master transcription factor for Th2 effector T cells, is associated with a poor prognosis [8, 50], demonstrating that a small subgroup of malignant Th2 cells exist, representing a more aggressive disease phenotype.

Both *FOXP1* and *HVEM* were found to be down-regulated in our analysis, and represent a disruption of T-cell maturation and the homeostasis of mature, effector T cells. FOXP1 is a member of the Forkhead box transcription factor family. *FOXP1*-deficient T cells display a premature activation phenotype in the thymus and the acquisition of effector functions. This indicates an improper generation of quiescent, naïve T-cells [51, 52]. Further, *HVEM*-deficient mice displayed a defect in the homeostasis of the long-term memory T-cell population and this defect is independent of either Th1/Th2 populations [35]. Histological evaluation of *FOXP1* in T-cell lymphomas has demonstrated that higher *FOXP1* expression leads to an improved overall survival in PTCL patients and may be associated with a less activated tumor phenotype [53]. However, *FOXP1* staining has proven to have disparate results [54, 55] and not all T-cell lymphomas have been found to be immunoreactive to FOXP1 [55].

*Thy-1* (CD90), a marker found to be upregulated in all mature T-cell lymphoma subtypes, is a marker for thymus-derived lymphocytes in mice [56], with additional implications for being a “cancer stem cell” marker present in both T- and B-malignancies due to its upregulation on immature cells [41, 57, 58]. Additionally, CD90 has been described as a consistent marker in different adult stem cell populations, reviewed in [59]. This observation is further supported by inverse relationships noted between *Thy-1* expression and T-cell differentiation or loss of stem-cell like potential [41, 60], with T-cell expression of *Thy-1* restricted to a small population of cortical thymocytes in humans, in addition to staining in T cells surrounding germinal centers [61]. In our immunohistochemical analysis, 46% (29/65) of T-cell lymphoma samples scored positive for CD90 lymphoid staining, with the staining being light and membranous. These results are in agreement with the above-discussed immature/undifferentiated phenotype observed in mature T-cell lymphomas, and suggest that these tumors may also increase the amount of immature T-cells present within the lymph node microenvironment.

Down-regulations are observed for many genes involved in TCR-signaling and the proximal events associated thereafter (*CD3G*, *ITK*, *LAT*, *PAG1*, *PDE4B*, *PRKCQ*). The attenuation of TCR/CD3 complex protein expression and proximal signaling molecules is featured as a hallmark of ALCL [62–64] with similarities noted in CD30+ PTCL-NOS cases [55]. These demarcations were inferred from a lack of immunoreactivity in PTCL tissue samples. Here, we have shown a down regulation of the mRNA of the aforementioned genes can be attributed to all subtypes of mature T-cell lymphomas studied, but with markedly lower expression for some genes in ALCL cases (Table 2). Further, ablations in TCR expression are seen with the down-regulation of *RICTOR*, the scaffolding molecule for mTORC2 signaling. mTORC2 signaling is vital for TCR surface expression, wherein *RICTOR*-deficient thymocytes displayed attenuated TCR surface expression [65]. The dysregulation of the mTORC2 pathway is further validated by the observed down-regulation of TCR components (*TRAC*, *TRBC1*) and stabilizing molecules (*TRAT1*) seen across multiple T-cell lymphoma subtypes (S1 Table).

For genes involving in apoptotic resistance and immune evasion, we observed a down-regulation of *IL23A*, and upregulation of *PSEN2*. IL23 is an immunomodulatory cytokine with controversial findings in terms of its anti-tumor properties [66, 67]. Despite this, IL23 has been described as effective in its activity against B-cell malignancies both *in vitro* and *in vivo* [68, 69]. Down-regulation of *IL23A* by T-cell malignancies represents a potential mechanism of immune evasion, which is further supported by an observed down-regulation of *IL12RB1* (seen in all subtypes except ALCL, S1 Table), a component of the IL23 receptor [70]. *PSEN2* is upregulated during some forms of apoptosis [71, 72], however its cleavage by caspase-3 (upregulated in all but AITL, S1 Table) results in a peptide fragment starting a negative feedback loop and inhibiting apoptosis [73]. These mechanisms of apoptotic avoidance represent potential therapeutic targets for further studies as α-IL23 has demonstrated activity against B-cell malignancies.

Using the top-scoring pairs method [18], the expression differential between *CAV1* and *BCL10* identified lymphoma samples with 98.4% sensitivity and 88.5% specificity. While the selection of *CAV1* and *BCL10* by the TSP method was *a priori*, the available genes were selected due to their differential expression, introducing bias. However, the *CAV1*/*BCL10* ratio is biologically relevant, with a prior inverse correlation observed in *CAV1* ^-/-^ T-lymphocytes [74], and BCL10 being downstream of CD26 signaling, of which CAV1 is the cognate ligand [75]. *BCL10* has been prior described as down-regulated among T-cell lymphomas, an observation in accordance with our findings. Despite this, the exact role for BCL10 is still inconclusive [39, 76, 77]. Our results demonstrate interplay between CAV1 and BCL10 in T-cell lymphomas and warrants further investigation.

In our analyses, *CAV1* was upregulated in each T-cell lymphoma subtype. Since *CAV1* is linked to disease progression and metastasis in different malignancies [15, 78–83], we sought to investigate the role of *CAV1* within the tumor microenvironment. PANTHER gene list analysis of *CAV1*-High versus *CAV1*-Low malignancies revealed an enrichment of multiple GO annotations for cytokine/chemokine activity, immune processes, and migration/motility within the *CAV1*-High group. The existence of the heterogeneity of *CAV1*expression in T-cell lymphomas was further solidified with our immunohistochemical results, with 55% (36/65) of tumor biopsies harboring lymphoid staining of CAV1. Taken together, these data suggest that T-cell malignancies with higher *CAV1* expression correlate with a GEP of possessing a more inflammatory tumor microenvironment and may hold more metastatic potential [78, 82, 83]. Further support for this is seen in the CAV1-protein networks determined by STRING. These networks displayed enrichment in proteins involved in cell migration and proliferation, indicating that CAV1 is directly involved in the aforementioned signaling processes and is an integral protein driving the differences within the *CAV1*-High and *CAV1*-Low tumor microenvironments. However, further analysis is required for confirmation of a role for CAV1 in disease progression in T-cell lymphomas. Chiefly, detailed research involving patient records and clinical characteristics is needed for a definitive conclusion. Such a study would be in-line with the current knowledge of *CAV1* expression within malignant entities, and is warranted based on the abovementioned results.

Interestingly, we also observed a long non-coding RNA (*LINC00273*) to be one of the highest genes differentially expressed between the CAV1-High and CAV1-Low groups. As long non-coding RNA signatures have been previously shown to be predictive in cancer [84–87], we chose to investigate whether this long non-coding RNA harbored any potential interactions with CAV1, given that CAV1 was the basis upon which sample stratification was conducted. Surprisingly, using computational prediction methods, we show that *LINC00273* is predicted to interact with CAV1 and members of a CAV1-interaction network in 3 out of 4 prediction metrics. The large discrepancy regarding RPISeq results could stem from the RPISeq-SVM having a lower accuracy in H. Sapiens non-coding RNA-protein interaction prediction (57.3%) as compared to RPISeq-RF (74.7%) [24]. While we observe a greater inconsistency in our data regarding prediction, both lncPro and RPIPred achieved a consensus prediction with the more accurate RPISeq-RF. Protein-RNA networks have been demonstrated as indispensible for cellular homeostasis [88–90], and the further investigation of long non-coding RNA signatures in T-cell lymphomas might hold predictive or therapeutic implications.

Herein, we present novel work concerning shared molecular underpinnings of five subtypes of mature T-cell lymphomas. These results enhance the knowledge of the basic biology of T-cell lymphomas, leading to insights concerning their activation and differentiation states, while also uncovering novel markers for future study. Finally, we characterize a role for *CAV1* within the tumor microenvironment, wherein higher *CAV1* expression among mature T-cell lymphomas coincides with a more inflammatory and invasive gene expression profile. Future study investigating the clinical implications of *CAV1* expression in mature T-cell lymphomas is warranted.

## Materials and Methods

### Ethics statement

All patient samples used within this study were from publicly available databases (i.e. gene expression samples) or commercial vendors (i.e. histology samples). Any and all patient disclosures were conducted within the original studies from which these samples were taken. The authors of this study state that they have neither tracing nor identification information that would disclose the identity of the patient samples herein.

### Data collation and normalization

Raw (.CEL) files were downloaded from the NCBI Gene Expression Omnibus (GEO) and loaded into BRB-ArrayTools using MAS5.0 normalization [91]. All samples were from publically available studies, as described in Table 1. In total, 52 healthy T-cell samples including: CD4^+^ T cells and CD8^+^ T cells taken from peripheral blood donors, and 187 non-treated, lymph node or splenic T-cell lymphoma biopsy samples were analyzed. All samples collated were prepared using HG-U133 plus 2 arrays. Replicate spots within arrays were averaged and multiple probes/probe sets were reduced to one per gene symbol using the maximally expressed probe (set) as measured by average intensity across arrays. Cell line samples, samples from non-lymphoid tissue, and samples not of interest to this study (e.g. B-cell samples and reactive tonsil samples) from the aforementioned datasets were discarded.

### T-cell compartment signature

To investigate the T-cell compartments of the malignant lymphoma samples, a “T-cell compartment signature” was created to be used in analyses of differential expression, thereby helping to reduce expression signatures from stromal components generated by processing whole tumor biopsies. This signature was compiled from GO annotation sets for T-cell receptor signaling, T-cell co-stimulation, T-cell homeostasis, and T-cell differentiation (http://geneontology.org/). Upon merging annotation lists, excluding duplicate genes, and manual curation for genes not expressed in T cells, 217 unique genes met these criteria (S4 Table).

### T-cell compartment differential expression analysis

For analysis of the differentially expressed genes between the T-cell compartment of malignant samples and healthy controls, the class comparison method among groups of arrays was used with the Multivariate Permutations test computed based on 1,000 random permutations. Genes were filtered according to the compiled T-cell compartment signature (see T-Cell Compartment Signature above) and results were deemed significant at p < 0.001, with a false discovery rate (FDR) of < 0.001. Each malignant subtype was analyzed separately followed by manual analysis of shared differentially expressed genes between each subtype as compared to healthy controls. Manual compilation allowed for the construction of a shared list of differentially expressed genes shared across each subtype.

### Establishment of a *CAV1-*expression threshold

To stratify malignant samples that overexpress *CAV1*, all samples were analyzed for *CAV1* expression via the 212097_at probe signal. A *CAV1* expression threshold was calculated by taking two standard deviations above the mean of the *CAV1* expression signal in healthy samples. Ninety-two percent (92%, 48/52) of healthy samples were below the calculated threshold, comprising 100% of non-outlier samples (identified as dots above the upper fence on the box-and-whisker plot). This threshold was selected as an adaptation of the 68-95-99.7 standard deviation short hand. While the healthy *CAV1* expression range did not follow a normal distribution, two standard deviations from the mean covered the abovementioned 92% of samples and the remaining 7% comprised outliers. This threshold was deemed acceptable as it classified all non-outlier samples within the healthy range and moving the threshold to three standard deviations exceeded all values within the healthy population. Malignant samples that were above the expression threshold were categorized as “*CAV1*-High”, while malignant samples below the expression threshold were categorized as “*CAV1*-Low”.

### *CAV1*-High versus *CAV1*-Low differential expression analysis

For analysis of the tumor microenvironment of *CAV1*-High and *CAV1*-Low samples, only genes with a significant (p < 0.01) correlation (“R” cut-off ± 0.22) to *CAV1* expression were included (n = 7,170) to reduce inter-malignancy variation. Differentially expressed genes (p < 0.001) were then determined using the class comparison among groups of arrays method with the Multivariate Permutations test computed based on 1,000 random permutations and a restriction on the proportion of false discoveries set at 1% with a confidence level of 99%.

### Gene list analysis of *CAV1*-High vs *CAV1*-Low

Fold-change values for genes found to be differentially expressed between the *CAV1*-High and *CAV1*-Low groups were input into the PANTHER gene analysis tool (PANTHER version 9.0; http://pantherdb.org/) [19, 20]. PANTHER enrichment and overrepresentation (excluding genes downregulated in *CAV1*-High) analyses were performed using the *Homo sapiens* reference list. Annotation data sets utilized included: GO molecular function, biological process, cellular component (complete versions), PANTHER protein class, and PANTHER pathways. Results were deemed significant at p < 0.05 using the Bonferroni correction method.

### CAV1-interaction networks

Genes upregulated in the *CAV1*-High and *CAV1*-Low expression groups were input separately into STRING v10 [92] to identify potential CAV1 protein-protein interaction networks. Interaction network constituents were determined by visual analysis of proteins shown to have direct or indirect interaction with CAV1. Enrichment analyses were conducted using the genome background and Bonferroni correction with results deemed significant at p < 0.001.

### Long non-coding RNA-protein interaction prediction

Long non-coding RNA-protein interaction (RPI) was predicted using three separate programs: lncPro [23], RPISeq [24], and RPI-Pred [25]. As RPISeq offers two prediction methods, a total of 4 distinct prediction outcomes were computed. RPI prediction software inputs were compiled according to program specificity. For lncPro, FASTA amino acid sequences for CAV1-interacting proteins, in addition to the FASTA nucleotide sequence for *LINC00273*, were input into the lncPro web-based platform (http://bioinfo.bjmu.edu.cn/lncpro/). The same protocol was used for the RPISeq web-based platform (http://pridb.gdcb.iastate.edu/RPISeq/). Equivocal information is required for the RPI-Pred platform (http://ctsb.is.wfubmc.edu/projects/rpi-pred/), in addition to protein block (PB) information [93]. As not all structures have been experimentally solved for the proteins of interest, the kPRED method [94] of PB structure prediction was used identical to Suresh *et al*. [25]. Per the literature, lncPro predictions were deemed positive for interaction scores ≥ 50, RPISeq for interaction scores ≥ 0.5, and RPI-Pred supplied text-based interpretations.

### Tissue microarray immunohistochemical staining

LY6161 high-density lymphoma and normal lymph node tissue arrays were ordered from U.S. Biomax, Inc and stained using the following protocol. Tissue array slides were deparaffinized and hydrated through xylene and a graded alcohol series, rinsed for 5 minutes in water, and incubated for 5 minutes in 3% H_2_O_2_. Slides were then washed 2 times followed by antigen unmasking using 1x antigen unmasking solution (Vector Laboratories) for 30 minutes. Slides were allowed to cool, washed 3 times in 0.1% Tween-20 PBS buffer, and blocked for 30 minutes with 2.5% normal horse blocking serum (Vector Laboratories). Slides were then stained with antibodies directed against BCL10 (1:100), CD90 (1:10), CAV1 (1:100), and GILZ (1:50), overnight at 4°C. Slides were subsequently washed 3 times, incubated for 30 minutes with Imm**PRESS** ^™^ reagent (Vector Laboratories), and washed an additional 3 times. Staining was revealed in peroxidase substrate DAB solution, with rinsing in tap water. Slides were counterstained with Hematoxylin QS (Vector Laboratories) and mounted with permanent mounting medium (Sigma).

### Immunohistochemical staining analysis

Stained slides were scanned using an iScan Coreo Au whole slide brightfield slide scanner (Ventana) and visualized using Ventana Image Viewer. Four independent researchers scored the slides based on the presence or absence of staining in lymphoid cells and/or the presence or absence of staining in stromal/non-lymphoid cells.

## Supporting Information

S1 Fig PANTHER enrichment analysis of the integrin signaling pathway. Molecule colors represent fold-change values (*CAV1*-High vs *CAV1*-Low): (dark blue = -3.361E00 to -1.536E00); (light blue = -1.536 to 1.542); (green = 1.542 to 1.715); (yellow = 1.715 to 1.882); (orange = 1.882 to 2.117); (red = 2.117 to 5.099).
(TIF)

S2 Fig PANTHER overrepresentation analysis for the PANTHER pathway: inflammation mediated by chemokine and cytokine signaling. Genes upregulated in *CAV1*-High samples as opposed to CAV1-Low are represented in red, while genes in the reference list are represented in gray.
(TIF)

S3 Fig PANTHER overrepresentation analysis for the PANTHER pathway: angiogenesis. Genes upregulated in *CAV1*-High samples as opposed to *CAV1*-Low are represented in red, while genes in the reference list are represented in gray.
(TIF)

S1 Table Genes significantly up- or down-regulated in at least 3/5 of T-cell lymphoma subtypes. (DOCX)

S2 Table Top-scoring pairs classification results. (DOCX)

S3 Table Detailed analysis of immunohistochemical staining on individual TMA samples. (DOCX)

S4 Table Genes comprising the T-cell compartment. (DOCX)
