# Correction: Conserved Molecular Underpinnings and Characterization of a Role for Caveolin-1 in the Tumor Microenvironment of Mature T-Cell Lymphomas

**DOI:** 10.1371/journal.pone.0146673

**Published:** 2016-01-05

**Authors:** Tyler A. Herek, Timothy D. Shew, Heather N. Spurgin, Christine E. Cutucache

In Table 2, the AITL fold-change value column was inadvertently duplicated into the PTCL-NOS fold-change column. Please see the corrected Table 2 here.

**Table 2:** Genes significantly up- or down-regulated across all mature T-cell lymphoma subtypes as compared with healthy CD4^+^ and CD8^+^ T-cell controls.

Gene Name	Symbol	Chromosomal Location	AITL Fold-Change	ALCL Fold-Change	ATLL Fold-Change	HSTL Fold-Change	PTCL-NOS Fold-Change
Caveolin-1	CAV1	7q31.1	16.33	12.52	38.17	34.41	17.03
Cyclin B2	CCNB2	15q22.2	7.55	8.93	13.99	9.65	10.81
Thy-1 cell surface antigen	THY1	11q23.3	13.65	12.98	9.46	2.46	9.88
Tumor necrosis factor receptor superfamily member 21	TNFRSF21	6p21.1	6.38	6.27	5.34	9.30	8.02
Enabled homolog	ENAH	1q42.12	5.62	5.36	2.52	22.63	5.32
Presenilin 2	PSEN2	1q42.13	2.94	4.77	2.26	3.39	3.09
3g molecule, gamma (CD3-TCR complex)	CD3G	11q23	-3.01	-6.81	-1.65	-2.02	-2.53
Tumor necrosis factor receptor superfamily member 14	TNFRSF14	1p36.32	-1.49	-1.58	-1.69	-1.99	-1.47
RPTOR independent companion of MTOR, complex 2	RICTOR	5p13.1	-2.42	-4.36	-1.75	-2.25	-2.03
Linker for activation of T-cells	LAT	16p11.2	-2.91	-3.61	-1.92	-3.76	-2.15
CD5 molecule	CD5	11q13	-4.12	-6.55	-2.10	-17.27	-3.98
Interleukin 23, alpha subunit p19	IL23A	12q13.3	-3.26	-3.50	-2.23	-4.51	-3.23
IL2-inducible T-cell kinase	ITK	5q31-q32	-3.67	-10.78	-2.36	-2.70	-3.82
Signal transducer and activator of transcription 5A	STAT5A	17q11.2	-2.14	-2.11	-2.50	-2.63	-2.27
TSC22 domain family, member 3	TSC22D3	Xq22.3	-5.66	-6.63	-2.56	-4.07	-4.99
Ubiquitin-associated and SH3 domain-containing protein A	UBASH3A	21q22.3	-2.43	-4.27	-2.86	-6.97	-2.46
Protein kinase C theta	PRKCQ	10p15	-2.98	-8.26	-3.58	-2.92	-3.86
Forkhead box P1	FOXP1	3p14.1	-4.90	-3.96	-3.69	-3.40	-3.84
Phosphoprotein membrane anchor with glycosphingolipid microdomains 1	PAG1	8q21.13	-3.81	-2.32	-4.30	-3.69	-2.63
B-cell CLL/lymphoma 10	BCL10	1p22	-4.96	-7.82	-6.54	-7.15	-6.26
Phosphodiesterase 4B, cAMP-specific	PDE4B	1p31	-4.21	-3.55	-7.44	-3.42	-2.45

AITL, angioimmunoblastic T-cell lymphoma; ALCL, anaplastic large cell lymphoma; ATLL, adult T-cell leukemia/lymphoma; HSTL, hepatosplenic T-cell lymphoma; PTCL-NOS, peripheral T-cell lymphoma, not otherwise specified
