# Supplementary material for: Conserved Molecular Underpinnings and Characterization of a Role for Caveolin-1 in the Tumor Microenvironment of Mature T-Cell Lymphomas
Source: PLoS One. 2015 Nov 13;10(11):e0142682. doi: 10.1371/journal.pone.0142682 (PMC4643970; doi:10.1371/journal.pone.0142682)
Supplement: S1 Table — (DOCX) [file pone.0142682.s004.docx]

| **Gene Name** | **Symbol** | **Chromosomal Location** | **OverUnder** | **N.S. in^1^** |
| --- | --- | --- | --- | --- |
| Programmed cell death 1 ligand 1 | CD274 | 9p24 | + | ATL, HSTL |
| B-cell CLL/lymphoma 3 | BCL3 | 19q13.1-q13.2 | + | ATL, HSTL |
| Dual specificity phosphatase 3 | DUSP3 | 17q21 | + | ATL |
| H2.0-like homeobox | HLX | 1q41 | + | HSTL |
| Interferon regulatory factor 4 | IRF4 | 6p25-p23 | + | HSTL |
| Lectin, galactoside-binding, soluble, 3 | LGALS3 | 14q22.3 | + | ATL, HSTL |
| Nck-associated protein 1-like | NCKAP1L | 12q13.1 | + | ALCL, ATL |
| p21 protein (Cdc42/Rac)-activated kinase 3 | PAK3 | Xq23 | + | HSTL |
| Programmed cell death 1 ligand 2 | PDCD1LG2 | 9p24.2 | + | ATL |
| phospholipase C, gamma 2 | PLCG2 | 16q24.1 | + | ATL |
| Ras-related C3 botulinum toxin substrate 1 | RAC1 | 7p22 | + | ALCL |
| Spinster homolog 2 | SPNS2 | 17p13.2 | + | ATL |
| SRC proto-oncogene, non-receptor tyrosine kinase | SRC | 20q12-q13 | + | ATL |
| Tumor necrosis factor ligand superfamily member 13B | TNFSF13B | 13q32-q34 | + | ATL, HSTL |
| Transforming growth factor beta-2 | TGFB2 | 1q41 | + | AITL, ATL |
| Caspase-3 | CASP3 | 4q34 | + | AITL |
| B-cell CLL/lymphoma 2 | BCL2 | 18q21.3 | - | ATL, HSTL |
| Butyrophilin subfamily 3 member A1 | BTN3A1 | 6p22.1 | - | ATL, HSTL |
| Caspase recruitment domain family, member 11 | CARD11 | 7p22 | - | ATL |
| C-C chemokine receptor type 7 | CCR7 | 17q12-q21.2 | - | ATL |
| T-cell surface glycoprotein CD3 zeta chain | CD247 | 1q24.2 | - | HSTL |
| T-cell-specific surface glycoprotein CD28 | CD28 | 2q33 | - | ATL |
| Dipeptidyl peptidase 4 | DPP4 | 2q24.3 | - | ALCL |
| ETS-related transcription factor Elf-1 | ELF1 | 13q13 | - | HSTL |
| FYN-binding protein | FYB | 5p13.1 | - | ATL |
| Inducible T-cell costimulator | ICOS | 2q33 | - | AITL, ATL |
| Interleukin 12 receptor, beta 1 | IL12RB1 | 19p13.1 | - | ALCL |
| Interleukin 27 receptor, alpha | IL27RA | 19p13.11 | - | AITL, ATL |
| LCK proto-oncogene, Src family tyrosine kinase | LCK | 1p34.3 | - | ATL, HSTL |
| Lymphoid enhancer-binding factor 1 | LEF1 | 4q23-q25 | - | ATL |
| Lectin, galactoside-binding, soluble, 8 | LGALS8 | 1q43 | - | HSTL |
| p21 protein (Cdc42/Rac)-activated kinase 2 | PAK2 | 3q29 | - | ATL |
| Pyruvate dehydrogenase kinase, isozyme 1 | PDK1 | 2q31.1 | - | ALCL, HSTL |
| Phosphatidylinositol 4,5-bisphosphate 3-kinase catalytic subunit delta isoform | PIK3CD | 1p36.2 | - | AITL, ATL |
| Phosphatidylinositol 3-kinase regulatory subunit alpha | PIK3R1 | 5q13.1 | - | ATL, HSTL |
| Phospholipase C, gamma 1 | PLCG1 | 20q12-q13.1 | - | ATL |
| Protein phosphatase 2, regulatory subunit B', gamma | PPP2R5C | 14q32.31 | - | ATL, HSTL |
| Prostaglandin E2 receptor EP4 subtype | PTGER4 | 5p13.1 | - | ATL, HSTL |
| Protein tyrosine phosphatase, receptor type, J | PTPRJ | 11p11.2 | - | ATL, HSTL |
| Ring finger and CCCH-type domains 1 | RC3H1 | 1q25.1 | - | ATL |
| RAR-related orphan receptor A | RORA | 15q22.2 | - | ATL |
| 40S ribosomal protein S6 | RPS6 | 9p21 | - | HSTL |
| Src kinase-associated phosphoprotein 1 | SKAP1 | 17q21.32 | - | ATL |
| Thymocyte expressed, positive selection associated 1 | TESPA1 | 12q13.2 | - | ATL |
| Thymocyte selection associated | THEMIS | 6q22.33 | - | HSTL |
| Transmembrane and immunoglobulin domain-containing protein 2 | TMIGD2 | 19p13.3 | - | HSTL |
| T-cell receptor alpha chain C region | TRAC | 14q11 | - | ATL |
| T-cell receptor-associated transmembrane adapter 1 | TRAT1 | 3q13 | - | HSTL |
| T-cell receptor beta-1 chain C region | TRBC1 | 7q34 | - | ATL |
| Tyrosine-protein kinase TXK | TXK | 4p12 | - | ATL |
| Ubiquitin A-52 residue ribosomal protein fusion product 1 | UBA52 | 19p13.1-p12 | - | ATL |
| Vav 1 guanine nucleotide exchange factor | VAV1 | 19p13.2 | - | ATL, HSTL |

^1^T-cell lymphoma subtypes for which the listed genes do not harbor significant differential expression
