# Supplementary material for: Conserved Molecular Underpinnings and Characterization of a Role for Caveolin-1 in the Tumor Microenvironment of Mature T-Cell Lymphomas
Source: PLoS One. 2015 Nov 13;10(11):e0142682. doi: 10.1371/journal.pone.0142682 (PMC4643970; doi:10.1371/journal.pone.0142682)
Supplement: S2 Table — (DOCX) [file pone.0142682.s005.docx]

| **Class** | **Sensitivity** | **Specificity** | **PPV^1^** | **NPV^2^** |
| --- | --- | --- | --- | --- |
| Healthy | 0.885 | 0.984 | 0.939 | 0.968 |
| Malignant | 0.984 | 0.885 | 0.968 | 0.939 |

^1^Positive Predictive Value (PPV) = n11/(n11+n21)

^2^Negative Predictive Value (NPV) = n22/(n12+n22)
