# Supplementary material for: Conserved Molecular Underpinnings and Characterization of a Role for Caveolin-1 in the Tumor Microenvironment of Mature T-Cell Lymphomas
Source: PLoS One. 2015 Nov 13;10(11):e0142682. doi: 10.1371/journal.pone.0142682 (PMC4643970; doi:10.1371/journal.pone.0142682)
Supplement: S3 Table — (DOCX) [file pone.0142682.s006.docx]

| **Diagnosis^1^** | **Sample ID^2^** | **Age^3^** | **Gender^4^** | **BCL10** | **CAV1** | **GILZ** | **CD90** |
| --- | --- | --- | --- | --- | --- | --- | --- |
| Anaplastic large cell lymphoma | B24/23 | 40 | F | **‡**** | **+** | **‡** | **-** |
| Anaplastic large cell lymphoma | S24/23 | 75 | F | **‡** | **‡** | **‡**** | **+** |
| Anaplastic large cell lymphoma | S26/25 | 76 | F | **-** | **+** | **+** | **-** |
| Anaplastic large cell lymphoma | S28/27 | 45 | M | **‡**** | **‡** | **‡** | **+** |
| Anaplastic large cell lymphoma | T2/1 | 60 | M | **‡** | **+** | **‡** | **‡** |
| Anaplastic large cell lymphoma | T4/3 | 71 | M | **‡** | **+** | **‡** | **‡** |
| Anaplastic large cell lymphoma | T6/5 | 21 | M | **‡** | **‡** | **‡** | **-** |
| Anaplastic large cell lymphoma | T8/7 | 23 | F | **‡** | **‡** | **‡** | **+** |
| Anaplastic large cell lymphoma | T10/9 | 40 | F | **‡** | **+** | **‡** | **‡** |
| Anaplastic large cell lymphoma | T12/11 | 64 | M | **‡** | **+** | **‡** | **‡** |
| Anaplastic large cell lymphoma | T14/13 | 40 | F | **‡** | **‡** | **‡** | **+** |
| Anaplastic large cell lymphoma | T16/15 | 45 | M | **‡** | **‡** | **‡** | **+** |
| Anaplastic large cell lymphoma | T18/17 | 34 | M | **‡** | **+** | **‡** | **‡** |
| Anaplastic large cell lymphoma | T20/19 | 52 | F | **‡** | **+** | **‡** | **+** |
| Angioimmunoblastic T-cell lymphoma | P8/7 | 64 | M | **‡** | **‡** | **‡** | **‡** |
| Angioimmunoblastic T-cell lymphoma | Q26/25 | 30 | M | **‡** | **‡** | **‡** | **-** |
| Angioimmunoblastic T-cell lymphoma | Q28/27 | 51 | M | **‡** | **‡** | **‡** | **‡** |
| Angioimmunoblastic T-cell lymphoma | R2/1 | 42 | F | **‡** | **‡** | **‡** | **‡** |
| Angioimmunoblastic T-cell lymphoma**^Δ^** | S8/7 | 55 | M | **‡** | **‡** | **‡**** | **‡** |
| Angioimmunoblastic T-cell lymphoma | S14/13 | 25 | F | **-** | **-** | **+**** | **-** |
| Angioimmunoblastic T-cell lymphoma | U22/21 | 49 | M | **‡** | **+** | **‡** | **‡** |
| Angioimmunoblastic T-cell lymphoma | U24/23 | 69 | F | **‡** | **+** | **‡** | **‡** |
| Peripheral T-cell lymphoma-NOS | O22/21 | 43 | M | **‡** | **‡** | **‡** | **-** |
| Peripheral T-cell lymphoma-NOS | O24/23 | 64 | M | **‡** | **+** | **‡** | **-** |
| Peripheral T-cell lymphoma-NOS | O26/25 | 52 | M | **‡** | **+** | **‡** | **-** |
| Peripheral T-cell lymphoma-NOS | O28/27 | 70 | M | **‡** | **‡** | **‡** | **‡** |
| Peripheral T-cell lymphoma-NOS | P4/3 | 41 | F | **‡**** | **+** | **‡**** | **-** |
| Peripheral T-cell lymphoma-NOS | P6/5 | 38 | M | **‡** | **‡** | **‡** | **‡** |
| Peripheral T-cell lymphoma-NOS | P10/9 | 71 | F | **‡** | **‡** | **‡** | **+** |
| Peripheral T-cell lymphoma-NOS | P1211 | 19 | M | **‡** | **‡** | **‡** | **‡** |
| Peripheral T-cell lymphoma-NOS | P14/13 | 17 | M | **‡** | **‡** | **‡** | **+** |
| Peripheral T-cell lymphoma-NOS | P16/15 | 51 | F | **‡** | **‡** | **‡** | **‡** |
| Peripheral T-cell lymphoma-NOS | P18/17 | 50 | F | **‡** | **‡** | **‡**** | **+** |
| Peripheral T-cell lymphoma-NOS | P20/19 | 45 | M | **‡** | **‡** | **‡** | **-** |
| Peripheral T-cell lymphoma-NOS | P22/21 | 41 | M | **‡** | **‡** | **‡** | **‡** |
| Peripheral T-cell lymphoma-NOS | P24/23 | 18 | M | **-** | **‡** | **‡** | **+** |
| Peripheral T-cell lymphoma-NOS | P26/25 | 43 | F | **-** | **‡** | **-** | **-** |
| Peripheral T-cell lymphoma-NOS | P28/27 | 40 | F | **‡** | **+** | **‡**** | **‡** |
| Peripheral T-cell lymphoma-NOS | Q2/1 | 65 | F | **‡** | **+** | **‡** | **+** |
| Peripheral T-cell lymphoma-NOS | Q4/3 | 54 | F | **‡** | **+** | **‡** | **+** |
| Peripheral T-cell lymphoma-NOS | Q6/5 | 57 | M | **‡** | **+** | **‡** | **-** |
| Peripheral T-cell lymphoma-NOS | Q8/7 | 36 | M | **‡** | **+** | **‡** | **-** |
| Peripheral T-cell lymphoma-NOS | Q14/13 | 53 | M | **‡** | **+** | **‡** | **‡** |
| Peripheral T-cell lymphoma-NOS | Q16/15 | 40 | F | **‡** | **+** | **‡** | **+** |
| Peripheral T-cell lymphoma-NOS | Q18/17 | 60 | F | **‡** | **‡** | **‡**** | **‡** |
| Peripheral T-cell lymphoma-NOS | Q20/19 | 22 | F | **‡** | **‡** | **‡** | **+** |
| Peripheral T-cell lymphoma-NOS | Q24/23 | 76 | M | **-** | **+** | **‡** | **+** |
| Peripheral T-cell lymphoma-NOS | R4/3 | 34 | M | **‡** | **‡** | **‡** | **‡** |
| Peripheral T-cell lymphoma-NOS | R6/5 | 58 | F | **‡** | **‡** | **‡** | **‡** |
| Peripheral T-cell lymphoma-NOS | R8/7 | 35 | F | **‡** | **+** | **‡**** | **+** |
| Peripheral T-cell lymphoma-NOS | R12/11 | 47 | M | **‡** | **+** | **-** | **+** |
| Peripheral T-cell lymphoma-NOS | R14/13 | 62 | F | **‡** | **‡** | **‡** | **‡** |
| Peripheral T-cell lymphoma-NOS | R16/15 | 36 | F | **‡** | **+** | **‡** | **+** |
| Peripheral T-cell lymphoma-NOS | R18/17 | 44 | F | **‡** | **+** | **‡**** | **‡** |
| Peripheral T-cell lymphoma-NOS | R20/19 | 37 | M | **‡** | **‡** | **‡** | **‡** |
| Peripheral T-cell lymphoma-NOS | R22/21 | 28 | M | **+**** | **‡** | **‡** | **-** |
| Peripheral T-cell lymphoma-NOS | R24/23 | 36 | M | **‡** | **+** | **‡** | **+** |
| Peripheral T-cell lymphoma-NOS | R26/25 | 63 | F | **‡** | **+** | **‡** | **-** |
| Peripheral T-cell lymphoma-NOS | R28/27 | 57 | M | **‡** | **‡** | **‡** | **-** |
| Peripheral T-cell lymphoma-NOS | S2/1 | 39 | F | **‡** | **‡** | **‡** | **‡** |
| Peripheral T-cell lymphoma-NOS | S4/3 | 13 | F | **‡** | **‡** | **‡** | **‡** |
| Peripheral T-cell lymphoma-NOS | S6/5 | 40 | F | **‡** | **‡** | **‡** | **‡** |
| Peripheral T-cell lymphoma-NOS | S10/9 | 32 | M | **‡** | **+** | **‡** | **‡** |
| Peripheral T-cell lymphoma-NOS | S12/11 | 22 | M | **‡** | **‡** | **‡** | **-** |
| Peripheral T-cell lymphoma-NOS | S16/15 | 12 | M | **‡** | **‡** | **‡**** | **‡** |
|  |  |  |  |  |  |  |  |

^1^All samples are of lymph node origin, unless otherwise denoted

^2^Sample ID is the location on the chip, followed by sample number (location, number)

^3^Age is reported in years

^4^Gender is reported as M (male) and F (female)

**‡**Staining observed in lymphoid cells

**+**Staining observed only in stromal/non-lymphoid cells

-No staining observed in sample

**Staining scored light in comparison to other positive samples

^Δ^Sample originating from splenic tissue
