# Supplementary material for: Conserved Molecular Underpinnings and Characterization of a Role for Caveolin-1 in the Tumor Microenvironment of Mature T-Cell Lymphomas
Source: PLoS One. 2015 Nov 13;10(11):e0142682. doi: 10.1371/journal.pone.0142682 (PMC4643970; doi:10.1371/journal.pone.0142682)
Supplement: S4 Table — (DOCX) [file pone.0142682.s007.docx]

| **ProbeSet** | **Name** | **Accession** | **UGCluster** | **Symbol** | **EntrezID** |
| --- | --- | --- | --- | --- | --- |
| 1552263_at | mitogen-activated protein kinase 1 | NM_138957 | Hs.431850 | MAPK1 | 5594 |
| 1552584_at | interleukin 12 receptor, beta 1 | NM_153701 | Hs.567294 | IL12RB1 | 3594 |
| 1552892_at | tumor necrosis factor receptor superfamily, member 13C | NM_052945 | Hs.344088 | TNFRSF13C | 115650 |
| 1552912_a_at | interleukin 23 receptor | NM_144701 | Hs.677426 | IL23R | 149233 |
| 1552995_at | interleukin 27 | NM_145659 | Hs.528111 | IL27 | 246778 |
| 1554078_s_at | DnaJ (Hsp40) homolog, subfamily A, member 3 | BC032100 | Hs.459779 | DNAJA3 | 9093 |
| 1555088_x_at | signal transducer and activator of transcription 5B | BC020868 | Hs.595276 | STAT5B | 6777 |
| 1557257_at | B-cell CLL/lymphoma 10 | AA994334 | Hs.193516 | BCL10 | 8915 |
| 1557783_at | MAP3K14 antisense RNA 1 | BC031942 | Hs.668927 | MAP3K14-AS1 | 100133991 |
| 1558687_a_at | forkhead box N1 | AI288186 | Hs.663679 | FOXN1 | 8456 |
| 1558972_s_at | thymocyte selection associated | BC043608 | Hs.661756 | THEMIS | 387357 |
| 1567035_at | chromosome 20 open reading frame 181 | U63828 | Hs.555583 | C20orf181 | 100128998 |
| 1567458_s_at | ras-related C3 botulinum toxin substrate 1 (rho family, small GTP binding protein Rac1) | AJ012502 | Hs.413812 | RAC1 | 5879 |
| 1568629_s_at | phosphoinositide-3-kinase, regulatory subunit 2 (beta) | BC033311 | Hs.371344 | PIK3R2 | 5296 |
| 1568943_at | inositol polyphosphate-5-phosphatase, 145kDa | BC027960 | Hs.262886 | INPP5D | 3635 |
| 200017_at | ribosomal protein S27a | NM_002954 | Hs.311640 | RPS27A | 6233 |
| 200081_s_at | ribosomal protein S6 | BE741754 | Hs.408073 | RPS6 | 6194 |
| 200633_at | ubiquitin B | NM_018955 | Hs.356190 | UBB | 7314 |
| 201105_at | lectin, galactoside-binding, soluble, 1 | NM_002305 | Hs.445351 | LGALS1 | 3956 |
| 201137_s_at | major histocompatibility complex, class II, DP beta 1 | NM_002121 | Hs.485130 | HLA-DPB1 | 3115 |
| 201300_s_at | prion protein | NM_000311 | Hs.472010 | PRNP | 5621 |
| 201332_s_at | signal transducer and activator of transcription 6, interleukin-4 induced | NM_003153 | Hs.524518 | STAT6 | 6778 |
| 201502_s_at | nuclear factor of kappa light polypeptide gene enhancer in B-cells inhibitor, alpha | AI078167 | Hs.81328 | NFKBIA | 4792 |
| 201523_x_at | ubiquitin-conjugating enzyme E2N | BE262760 | Hs.524630 | UBE2N | 7334 |
| 201536_at | dual specificity phosphatase 3 | AL048503 | Hs.181046 | DUSP3 | 1845 |
| 201877_s_at | protein phosphatase 2, regulatory subunit B', gamma | NM_002719 | Hs.368264 | PPP2R5C | 5527 |
| 202178_at | protein kinase C, zeta | NM_002744 | Hs.496255 | PRKCZ | 5590 |
| 202205_at | vasodilator-stimulated phosphoprotein | NM_003370 | Hs.515469 | VASP | 7408 |
| 202288_at | mechanistic target of rapamycin (serine/threonine kinase) | U88966 | Hs.338207 | MTOR | 2475 |
| 202329_at | c-src tyrosine kinase | NM_004383 | Hs.77793 | CSK | 1445 |
| 202432_at | protein phosphatase 3, catalytic subunit, beta isozyme | NM_021132 | Hs.500067 | PPP3CB | 5532 |
| 202535_at | Fas (TNFRSF6)-associated via death domain | NM_003824 | Hs.86131 | FADD | 8772 |
| 202625_at | v-yes-1 Yamaguchi sarcoma viral related oncogene homolog | AI356412 | Hs.491767 | LYN | 4067 |
| 202705_at | cyclin B2 | NM_004701 | Hs.194698 | CCNB2 | 9133 |
| 202763_at | caspase 3, apoptosis-related cysteine peptidase | NM_004346 | Hs.141125 | CASP3 | 836 |
| 202789_at | phospholipase C, gamma 1 | AL022394 | Hs.268177 | PLCG1 | 5335 |
| 202820_at | aryl hydrocarbon receptor | NM_001621 | Hs.171189 | AHR | 196 |
| 202932_at | v-yes-1 Yamaguchi sarcoma viral oncogene homolog 1 | NM_005433 | Hs.194148 | YES1 | 7525 |
| 203010_at | signal transducer and activator of transcription 5A | NM_003152 | Hs.437058 | STAT5A | 6776 |
| 203140_at | B-cell CLL/lymphoma 6 | NM_001706 | Hs.478588 | BCL6 | 604 |
| 203233_at | interleukin 4 receptor | NM_000418 | Hs.513457 | IL4R | 3566 |
| 203290_at | major histocompatibility complex, class II, DQ alpha 1 | NM_002122 | Hs.387679 | HLA-DQA1 | 3117 |
| 203332_s_at | inositol polyphosphate-5-phosphatase, 145kDa | NM_005541 | Hs.262886 | INPP5D | 3635 |
| 203547_at | CD4 molecule | U47924 | Hs.631659 | CD4 | 920 |
| 203685_at | B-cell CLL/lymphoma 2 | NM_000633 | Hs.150749 | BCL2 | 596 |
| 203708_at | phosphodiesterase 4B, cAMP-specific | NM_002600 | Hs.198072 | PDE4B | 5142 |
| 203717_at | dipeptidyl-peptidase 4 | NM_001935 | Hs.368912 | DPP4 | 1803 |
| 203749_s_at | retinoic acid receptor, alpha | AI806984 | Hs.654583 | RARA | 5914 |
| 203879_at | phosphatidylinositol-4,5-bisphosphate 3-kinase, catalytic subunit delta | U86453 | Hs.518451 | PIK3CD | 5293 |
| 204285_s_at | phorbol-12-myristate-13-acetate-induced protein 1 | AI857639 | Hs.96 | PMAIP1 | 5366 |
| 204369_at | phosphatidylinositol-4,5-bisphosphate 3-kinase, catalytic subunit alpha | NM_006218 | Hs.553498 | PIK3CA | 5290 |
| 204401_at | potassium intermediate/small conductance calcium-activated channel, subfamily N, member 4 | NM_002250 | Hs.10082 | KCNN4 | 3783 |
| 204491_at | phosphodiesterase 4D, cAMP-specific | R40917 | Hs.117545 | PDE4D | 5144 |
| 204524_at | 3-phosphoinositide dependent protein kinase-1 | NM_002613 | Hs.459691 | PDPK1 | 5170 |
| 204562_at | interferon regulatory factor 4 | NM_002460 | Hs.401013 | IRF4 | 3662 |
| 204613_at | phospholipase C, gamma 2 (phosphatidylinositol-specific) | NM_002661 | Hs.372303 | PLCG2 | 5336 |
| 204639_at | adenosine deaminase | NM_000022 | Hs.654536 | ADA | 100 |
| 204725_s_at | NCK adaptor protein 1 | NM_006153 | Hs.126889 | NCK1 | 4690 |
| 204780_s_at | Fas cell surface death receptor | AA164751 | Hs.244139 | FAS | 355 |
| 204798_at | v-myb avian myeloblastosis viral oncogene homolog | NM_005375 | Hs.606320 | MYB | 4602 |
| 204891_s_at | lymphocyte-specific protein tyrosine kinase | NM_005356 | Hs.470627 | LCK | 3932 |
| 204897_at | prostaglandin E receptor 4 (subtype EP4) | AA897516 | Hs.199248 | PTGER4 | 5734 |
| 204908_s_at | B-cell CLL/lymphoma 3 | NM_005178 | Hs.31210 | BCL3 | 602 |
| 205027_s_at | mitogen-activated protein kinase kinase kinase 8 | NM_005204 | Hs.432453 | MAP3K8 | 1326 |
| 205198_s_at | ATPase, Cu++ transporting, alpha polypeptide | NM_000052 | Hs.496414 | ATP7A | 538 |
| 205205_at | v-rel avian reticuloendotheliosis viral oncogene homolog B | NM_006509 | Hs.654402 | RELB | 5971 |
| 205207_at | interleukin 6 (interferon, beta 2) | NM_000600 | Hs.654458 | IL6 | 3569 |
| 205269_at | lymphocyte cytosolic protein 2 (SH2 domain containing leukocyte protein of 76kDa) | AI123251 | Hs.304475 | LCP2 | 3937 |
| 205456_at | CD3e molecule, epsilon (CD3-TCR complex) | NM_000733 | Hs.3003 | CD3E | 916 |
| 205504_at | Bruton agammaglobulinemia tyrosine kinase | NM_000061 | Hs.159494 | BTK | 695 |
| 205558_at | TNF receptor-associated factor 6, E3 ubiquitin protein ligase | NM_004620 | Hs.444172 | TRAF6 | 7189 |
| 205681_at | BCL2-related protein A1 | NM_004049 | Hs.227817 | BCL2A1 | 597 |
| 205790_at | src kinase associated phosphoprotein 1 | NM_003726 | Hs.316931 | SKAP1 | 8631 |
| 205965_at | basic leucine zipper transcription factor, ATF-like | NM_006399 | Hs.509964 | BATF | 10538 |
| 206056_x_at | sialophorin | X52075 | Hs.632188 | SPN | 6693 |
| 206060_s_at | protein tyrosine phosphatase, non-receptor type 22 (lymphoid) | NM_015967 | Hs.535276 | PTPN22 | 26191 |
| 206219_s_at | vav 1 guanine nucleotide exchange factor | NM_005428 | Hs.116237 | VAV1 | 7409 |
| 206301_at | tec protein tyrosine kinase | NM_003215 | Hs.479670 | TEC | 7006 |
| 206337_at | chemokine (C-C motif) receptor 7 | NM_001838 | Hs.370036 | CCR7 | 1236 |
| 206419_at | RAR-related orphan receptor C | NM_005060 | Hs.256022 | RORC | 6097 |
| 206464_at | BMX non-receptor tyrosine kinase | NM_001721 | Hs.495731 | BMX | 660 |
| 206545_at | CD28 molecule | NM_006139 | Hs.443123 | CD28 | 940 |
| 206591_at | recombination activating gene 1 | NM_000448 | Hs.73958 | RAG1 | 5896 |
| 206618_at | interleukin 18 receptor 1 | NM_003855 | Hs.469521 | IL18R1 | 8809 |
| 206687_s_at | protein tyrosine phosphatase, non-receptor type 6 | NM_002831 | Hs.63489 | PTPN6 | 5777 |
| 206804_at | CD3g molecule, gamma (CD3-TCR complex) | NM_000073 | Hs.2259 | CD3G | 917 |
| 206828_at | TXK tyrosine kinase | NM_003328 | Hs.479669 | TXK | 7294 |
| 206854_s_at | mitogen-activated protein kinase kinase kinase 7 | NM_003188 | Hs.594838 | MAP3K7 | 6885 |
| 206978_at | chemokine (C-C motif) receptor 2 | NM_000647 | Hs.511794 | CCR2 | 729230 |
| 206999_at | interleukin 12 receptor, beta 2 | NM_001559 | Hs.479347 | IL12RB2 | 3595 |
| 207091_at | purinergic receptor P2X, ligand-gated ion channel, 7 | NM_002562 | Hs.729169 | P2RX7 | 5027 |
| 207160_at | interleukin 12A (natural killer cell stimulatory factor 1, cytotoxic lymphocyte maturation factor 1, p35) | NM_000882 | Hs.673 | IL12A | 3592 |
| 207163_s_at | v-akt murine thymoma viral oncogene homolog 1 | NM_005163 | Hs.525622 | AKT1 | 207 |
| 207426_s_at | tumor necrosis factor (ligand) superfamily, member 4 | NM_003326 | Hs.181097 | TNFSF4 | 7292 |
| 207538_at | interleukin 4 | NM_000589 | Hs.73917 | IL4 | 3565 |
| 207571_x_at | thymocyte selection associated family member 2 | NM_004848 | Hs.10649 | THEMIS2 | 9473 |
| 207634_at | programmed cell death 1 | NM_005018 | Hs.158297 | PDCD1 | 5133 |
| 207782_s_at | presenilin 1 | NM_007319 | Hs.3260 | PSEN1 | 5663 |
| 207849_at | interleukin 2 | NM_000586 | Hs.89679 | IL2 | 3558 |
| 207907_at | tumor necrosis factor (ligand) superfamily, member 14 | NM_003807 | Hs.129708 | TNFSF14 | 8740 |
| 208377_s_at | calcium channel, voltage-dependent, L type, alpha 1F subunit | NM_005183 | Hs.632799 | CACNA1F | 778 |
| 208478_s_at | BCL2-associated X protein | NM_004324 | Hs.624291 | BAX | 581 |
| 208727_s_at | cell division cycle 42 | BC002711 | Hs.467637 | CDC42 | 998 |
| 208763_s_at | TSC22 domain family, member 3 | AL110191 | Hs.522074 | TSC22D3 | 1831 |
| 208851_s_at | Thy-1 cell surface antigen | AL161958 | Hs.644697 | THY1 | 7070 |
| 208877_at | p21 protein (Cdc42/Rac)-activated kinase 2 | W74494 | Hs.518530 | PAK2 | 5062 |
| 208894_at | major histocompatibility complex, class II, DR alpha | M60334 | Hs.520048 | HLA-DRA | 3122 |
| 208933_s_at | lectin, galactoside-binding, soluble, 8 | AI659005 | Hs.4082 | LGALS8 | 3964 |
| 208949_s_at | lectin, galactoside-binding, soluble, 3 | BC001120 | Hs.531081 | LGALS3 | 3958 |
| 209083_at | coronin, actin binding protein, 1A | U34690 | Hs.415067 | CORO1A | 11151 |
| 209239_at | nuclear factor of kappa light polypeptide gene enhancer in B-cells 1 | M55643 | Hs.618430 | NFKB1 | 4790 |
| 209341_s_at | inhibitor of kappa light polypeptide gene enhancer in B-cells, kinase beta | AU153366 | Hs.597664 | IKBKB | 3551 |
| 209354_at | tumor necrosis factor receptor superfamily, member 14 | BC002794 | Hs.512898 | TNFRSF14 | 8764 |
| 209498_at | carcinoembryonic antigen-related cell adhesion molecule 1 (biliary glycoprotein) | X16354 | Hs.512682 | CEACAM1 | 634 |
| 209545_s_at | receptor-interacting serine-threonine kinase 2 | AF064824 | Hs.103755 | RIPK2 | 8767 |
| 209604_s_at | GATA binding protein 3 | BC003070 | Hs.524134 | GATA3 | 2625 |
| 209648_x_at | suppressor of cytokine signaling 5 | AL136896 | Hs.468426 | SOCS5 | 9655 |
| 209666_s_at | conserved helix-loop-helix ubiquitous kinase | AF080157 | Hs.198998 | CHUK | 1147 |
| 209670_at | T cell receptor alpha constant | M12959 | Hs.74647 | TRAC | 28755 |
| 209682_at | Cbl proto-oncogene B, E3 ubiquitin protein ligase | U26710 | Hs.430589 | CBLB | 868 |
| 209728_at | major histocompatibility complex, class II, DR beta 4 | BC005312 | Hs.534322 | HLA-DRB4 | 3126 |
| 209734_at | NCK-associated protein 1-like | BC001604 | Hs.182014 | NCKAP1L | 3071 |
| 209770_at | butyrophilin, subfamily 3, member A1 | U90552 | Hs.191510 | BTN3A1 | 11119 |
| 209929_s_at | inhibitor of kappa light polypeptide gene enhancer in B-cells, kinase gamma | AF091453 | Hs.43505 | IKBKG | 8517 |
| 209933_s_at | CD300a molecule | AF020314 | Hs.9688 | CD300A | 11314 |
| 210018_x_at | mucosa associated lymphoid tissue lymphoma translocation gene 1 | AB026118 | Hs.601217 | MALT1 | 10892 |
| 210031_at | CD247 molecule | J04132 | Hs.156445 | CD247 | 919 |
| 210038_at | protein kinase C, theta | AL137145 | Hs.498570 | PRKCQ | 5588 |
| 210284_s_at | TGF-beta activated kinase 1/MAP3K7 binding protein 2 | AF241230 | Hs.269775 | TAB2 | 23118 |
| 210439_at | inducible T-cell co-stimulator | AB023135 | Hs.56247 | ICOS | 29851 |
| 210792_x_at | SIVA1, apoptosis-inducing factor | AF033111 | Hs.112058 | SIVA1 | 10572 |
| 211005_at | linker for activation of T cells | AF036906 | Hs.632179 | LAT | 27040 |
| 211009_s_at | zinc finger protein 271 | AF159567 | Hs.314246 | ZNF271 | 10778 |
| 211296_x_at | ubiquitin C | AB009010 | Hs.520348 | UBC | 7316 |
| 211339_s_at | IL2-inducible T-cell kinase | D13720 | Hs.558348 | ITK | 3702 |
| 211373_s_at | presenilin 2 | U34349 | Hs.25363 | PSEN2 | 5664 |
| 211654_x_at | major histocompatibility complex, class II, DQ beta 1 | M17565 | Hs.409934 | HLA-DQB1 | 3119 |
| 211795_s_at | FYN binding protein | AF198052 | Hs.370503 | FYB | 2533 |
| 212097_at | caveolin 1, caveolae protein, 22kDa | AU147399 | Hs.74034 | CAV1 | 857 |
| 212240_s_at | phosphoinositide-3-kinase, regulatory subunit 1 (alpha) | AI679268 | Hs.132225 | PIK3R1 | 5295 |
| 212420_at | E74-like factor 1 (ets domain transcription factor) | AL559590 | Hs.135646 | ELF1 | 1997 |
| 212588_at | protein tyrosine phosphatase, receptor type, C | Y00062 | Hs.654514 | PTPRC | 5788 |
| 212610_at | protein tyrosine phosphatase, non-receptor type 11 | U79291 | Hs.506852 | PTPN11 | 5781 |
| 212646_at | raftlin, lipid raft linker 1 | D42043 | Hs.98910 | RFTN1 | 23180 |
| 212688_at | phosphatidylinositol-4,5-bisphosphate 3-kinase, catalytic subunit beta | BC003393 | Hs.239818 | PIK3CB | 5291 |
| 213136_at | protein tyrosine phosphatase, non-receptor type 2 | AI828880 | Hs.654527 | PTPN2 | 5771 |
| 213193_x_at | T cell receptor beta constant 1 | AL559122 | Hs.382212 | TRBC1 | 28639 |
| 213324_at | v-src avian sarcoma (Schmidt-Ruppin A-2) viral oncogene homolog | AK024281 | Hs.195659 | SRC | 6714 |
| 213450_s_at | inducible T-cell co-stimulator ligand | AI659611 | Hs.14155 | ICOSLG | 23308 |
| 213539_at | CD3d molecule, delta (CD3-TCR complex) | NM_000732 | Hs.504048 | CD3D | 915 |
| 214228_x_at | tumor necrosis factor receptor superfamily, member 4 | AJ277151 | Hs.129780 | TNFRSF4 | 7293 |
| 214438_at | H2.0-like homeobox | M60721 | Hs.74870 | HLX | 3142 |
| 214607_at | p21 protein (Cdc42/Rac)-activated kinase 3 | AW085556 | Hs.593599 | PAK3 | 5063 |
| 215416_s_at | stomatin (EPB72)-like 2 | AC004472 | Hs.3439 | STOML2 | 30968 |
| 216033_s_at | FYN oncogene related to SRC, FGR, YES | S74774 | Hs.390567 | FYN | 2534 |
| 216379_x_at | CD24 molecule | AK000168 | Hs.644105 | CD24 | 100133941 |
| 217147_s_at | T cell receptor associated transmembrane adaptor 1 | AJ240085 | Hs.138701 | TRAT1 | 50852 |
| 217808_s_at | mitogen-activated protein kinase associated protein 1 | NM_024117 | Hs.495138 | MAPKAP1 | 79109 |
| 217820_s_at | enabled homolog (Drosophila) | NM_018212 | Hs.497893 | ENAH | 55740 |
| 218845_at | dual specificity phosphatase 22 | NM_020185 | Hs.29106 | DUSP22 | 56940 |
| 218856_at | tumor necrosis factor receptor superfamily, member 21 | NM_016629 | Hs.443577 | TNFRSF21 | 27242 |
| 219259_at | sema domain, immunoglobulin domain (Ig), transmembrane domain (TM) and short cytoplasmic domain, (semaphorin) 4A | NM_022367 | Hs.408846 | SEMA4A | 64218 |
| 220049_s_at | programmed cell death 1 ligand 2 | NM_025239 | Hs.532279 | PDCD1LG2 | 80380 |
| 220054_at | interleukin 23, alpha subunit p19 | NM_016584 | Hs.98309 | IL23A | 51561 |
| 220418_at | ubiquitin associated and SH3 domain containing A | NM_018961 | Hs.473912 | UBASH3A | 53347 |
| 220587_s_at | MTOR associated protein, LST8 homolog (S. cerevisiae) | NM_022372 | Hs.29203 | MLST8 | 64223 |
| 220812_s_at | HERV-H LTR-associating 2 | NM_007072 | Hs.252351 | HHLA2 | 11148 |
| 221334_s_at | forkhead box P3 | NM_014009 | Hs.247700 | FOXP3 | 50943 |
| 221457_s_at | butyrophilin-like 2 (MHC class II associated) | NM_019602 | Hs.534471 | BTNL2 | 56244 |
| 221558_s_at | lymphoid enhancer-binding factor 1 | AF288571 | Hs.743478 | LEF1 | 51176 |
| 221700_s_at | ubiquitin A-52 residue ribosomal protein fusion product 1 | AF348700 | Hs.5308 | UBA52 | 7311 |
| 221827_at | RanBP-type and C3HC4-type zinc finger containing 1 | BE788439 | Hs.247280 | RBCK1 | 10616 |
| 222062_at | interleukin 27 receptor, alpha | AI983115 | Hs.132781 | IL27RA | 9466 |
| 222920_s_at | thymocyte expressed, positive selection associated 1 | BG231515 | Hs.33187 | TESPA1 | 9840 |
| 223049_at | growth factor receptor-bound protein 2 | AF246238 | Hs.444356 | GRB2 | 2885 |
| 223207_x_at | phosphohistidine phosphatase 1 | AF285119 | Hs.409834 | PHPT1 | 29085 |
| 223502_s_at | tumor necrosis factor (ligand) superfamily, member 13b | AF134715 | Hs.525157 | TNFSF13B | 10673 |
| 223506_at | zinc finger CCCH-type containing 8 | AF334161 | Hs.418416 | ZC3H8 | 84524 |
| 223514_at | caspase recruitment domain family, member 11 | AF322641 | Hs.648101 | CARD11 | 84433 |
| 223816_at | solute carrier family 46, member 2 | AF242557 | Hs.512668 | SLC46A2 | 57864 |
| 223917_s_at | solute carrier family 39 (zinc transporter), member 3 | BC000085 | Hs.515046 | SLC39A3 | 29985 |
| 224838_at | forkhead box P1 | AK026898 | Hs.59368 | FOXP1 | 27086 |
| 225424_at | glycerol-3-phosphate acyltransferase, mitochondrial | AB046780 | Hs.42586 | GPAM | 57678 |
| 225606_at | BCL2-like 11 (apoptosis facilitator) | AI949179 | Hs.469658 | BCL2L11 | 10018 |
| 225626_at | phosphoprotein associated with glycosphingolipid microdomains 1 | AK000680 | Hs.266175 | PAG1 | 55824 |
| 225671_at | spinster homolog 2 (Drosophila) | AL568674 | Hs.22824 | SPNS2 | 124976 |
| 225893_at | ring finger and CCCH-type domains 1 | AL589593 | Hs.30258 | RC3H1 | 149041 |
| 226223_at | PRKC, apoptosis, WT1, regulator | AI091432 | Hs.643130 | PAWR | 5074 |
| 226312_at | RPTOR independent companion of MTOR, complex 2 | BF940270 | Hs.407926 | RICTOR | 253260 |
| 226507_at | p21 protein (Cdc42/Rac)-activated kinase 1 | AU154408 | Hs.435714 | PAK1 | 5058 |
| 226682_at | RAR-related orphan receptor A | AW006185 | Hs.560343 | RORA | 6095 |
| 227216_at | RGD motif, leucine rich repeats, tropomodulin domain and proline-rich containing | AI560765 | Hs.611432 | RLTPR | 146206 |
| 227396_at | protein tyrosine phosphatase, receptor type, J | AI631833 | Hs.318547 | PTPRJ | 5795 |
| 227458_at | CD274 molecule | AI608902 | Hs.521989 | CD274 | 29126 |
| 227677_at | Janus kinase 3 | BF512748 | Hs.515247 | JAK3 | 3718 |
| 227822_at | zinc finger protein 605 | AI341321 | Hs.29698 | ZNF605 | 100289635 |
| 228121_at | transforming growth factor, beta 2 | AU145950 | Hs.133379 | TGFB2 | 7042 |
| 228139_at | receptor-interacting serine-threonine kinase 3 | NM_006871 | Hs.268551 | RIPK3 | 11035 |
| 229846_s_at | mitogen-activated protein kinase associated protein 1 | BF939919 | Hs.495138 | MAPKAP1 | 79109 |
| 230202_at | v-rel avian reticuloendotheliosis viral oncogene homolog A | AI703057 | Hs.502875 | RELA | 5970 |
| 230489_at | CD5 molecule | AI797836 | Hs.58685 | CD5 | 921 |
| 231017_at | serine/threonine kinase 11 | AI914604 | Hs.515005 | STK11 | 6794 |
| 231635_x_at | ring finger protein 31 | AW301351 | Hs.375217 | RNF31 | 55072 |
| 231716_at | ring finger and CCCH-type domains 2 | AF255304 | Hs.533499 | RC3H2 | 54542 |
| 235557_at | glycerol-3-phosphate acyltransferase 2, mitochondrial | AW082827 | Hs.348629 | GPAT2 | 150763 |
| 236226_at | B and T lymphocyte associated | AW294080 | Hs.445162 | BTLA | 151888 |
| 236341_at | cytotoxic T-lymphocyte-associated protein 4 | AI733018 | Hs.247824 | CTLA4 | 1493 |
| 238725_at | interferon regulatory factor 1 | AW392551 | Hs.436061 | IRF1 | 3659 |
| 239188_at | protein phosphatase 2, regulatory subunit B'', gamma | AA838428 | Hs.530712 | PPP2R3C | 55012 |
| 239294_at | phosphatidylinositol-4,5-bisphosphate 3-kinase, catalytic subunit gamma | AA810265 | Hs.32942 | PIK3CG | 5294 |
| 242282_at | zinc finger protein, FOG family member 1 | AI889717 | Hs.632218 | ZFPM1 | 161882 |
| 242622_x_at | phosphatase and tensin homolog | T86737 | Hs.500466 | PTEN | 5728 |
| 243244_at | calcium channel, voltage-dependent, beta 4 subunit | H22005 | Hs.120725 | CACNB4 | 785 |
| 244716_x_at | transmembrane and immunoglobulin domain containing 2 | AI817976 | Hs.263928 | TMIGD2 | 126259 |
| 34726_at | calcium channel, voltage-dependent, beta 3 subunit | U07139 | Hs.250712 | CACNB3 | 784 |
| 38269_at | protein kinase D2 | AL050147 | Hs.466987 | PRKD2 | 25865 |
| 38964_r_at | Wiskott-Aldrich syndrome | U12707 | Hs.2157 | WAS | 7454 |
